# Catalytic Asymmetric Formal Total Synthesis of (−)-Triptophenolide and (+)-Triptolide

**DOI:** 10.1007/s13659-016-0100-z

**Published:** 2016-04-20

**Authors:** Wen-Dan Xu, Liang-Qun Li, Ming-Ming Li, Hui-Chun Geng, Hong-Bo Qin

**Affiliations:** 1 State Key Laboratory of Photochemistry and Plant Resources in West China, Kunming Institute of Botany, Chinese Academy of Sciences, Kunming, 650201 Yunnan People’s Republic of China; 2 University of Chinese Academy of Sciences, Beijing, 100049 People’s Republic of China; 3 Yunnan Baiyao Group Corporation Limited, Kunming, 650032 People’s Republic of China

**Keywords:** Total synthesis, Catalytic asymmetric, Triptophenolide, Triptolide

## Abstract

**Abstract:**

Catalytic asymmetric formal synthesis of (−)-Triptophenolide and (+)-Triptolide have been achieved. Key reaction involves Palladium catalyzed asymmetric conjugate addition of aryl boronic acid to 3-methyl cyclohexe-1-none to form quaternary carbon. Claisen rearrangement and subsequent aldol reaction furnished *trans*-decaline key intermediate, which assured a formal total synthesis of (−)-Triptophenolide and (+)-Triptolide.

**Graphical Abstract:**

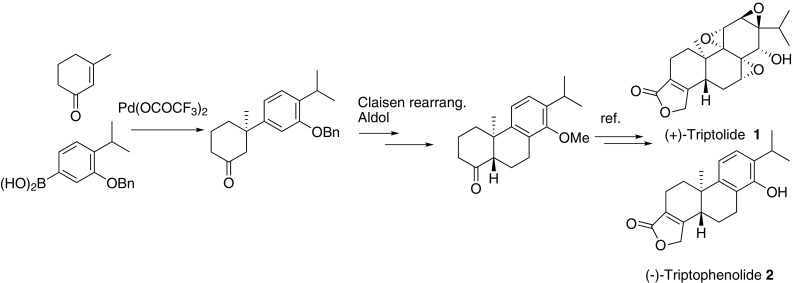

**Electronic supplementary material:**

The online version of this article (doi:10.1007/s13659-016-0100-z) contains supplementary material, which is available to authorized users.

## Introduction

**Fig. 1:**
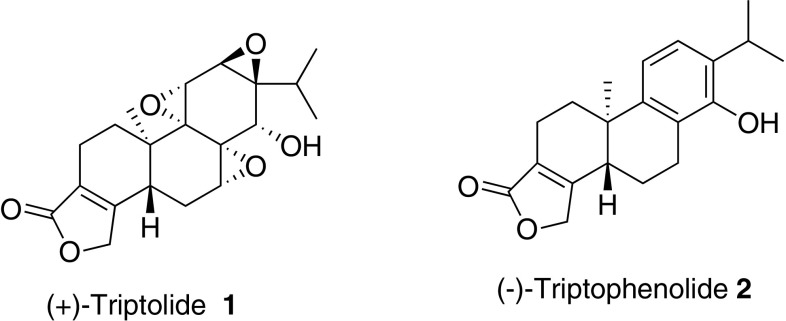
Structures of compounds **1**–**2**

*Tripterygium wilfordii* Hook F has long been used to treat rheumatoid arthritis in southern China. The active component was first determined to be Triptolide (TP) (Fig. 1) after it was isolated by Kupchan in 1972 [1]. The unique tri-epoxide moiety attracted numerous attention to identify its molecular mechanism of action in cancer [2–8], immunosuppression [9–11] and contraception [12]. Triptophenolide, a precursor of TP, exhibited potent anti inflammatory and immunosuppressive activity [13, 14].

By using TP as tool molecule, biological researches have accumulated a lot of discoveries which keep TP as a hot topic. These findings have, in return, promoted the development of efficient synthesis strategy. We seek to discover new lead compounds, especially from nature terpenoids, for the development of anticancer drug [15–17]. Hence, TP is a valuable target for us to investigate SAR of tri-epoxide. We initiated this research in order to develop a catalytic asymmetric synthetic route because it has not been achieved.

Reports of its total synthesis were available from 1980, [18, 19] and the maximum productivity was reached around 2000 [20–23]. So far, only two stereoselective synthesis were reported, and both used chiral auxiliaries [22, 24]. On the other hand, in 2008, Sherburn reported an elegant synthesis with a Diels–Alder reaction [25]. Only preliminary enantioselective synthesis of vinyl iodide was performed and we regard it as a racemic synthesis and will report the first catalytic asymmetric formal total synthesis of (−)-Triptophenolide and (+)-TP, which features a palladium catalyzed conjugate addition of arylboronic acid to cyclic enone to construct all-carbon quaternary stereocenter, followed by new cyclization strategy involving claisen rearrangement [26] and aldol reaction.

**Scheme 1:**
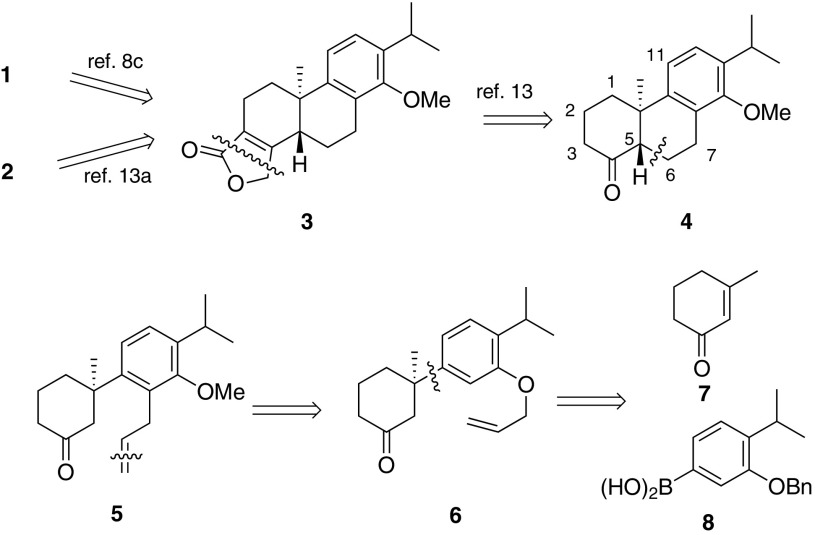
Retrosynthetic analysis of **1** and **2**

The retrosynthetic analysis is depicted in Scheme 1. Unlike precedent methods including biogenetic cascade radical [21, 27] or cationic [28–30] cyclization and alkylation of benzocyclohexanone derivatives, [18, 24] or Diels–Alder reaction [25] to construct the quaternary center, we envisioned that it could be formed enantioselectively via arylboronic acid addition to cyclic enone, a methodology pioneered by Prof. Lu [31] and developed by Prof. Stoltz [32–34].

As illustrated in scheme 1, TP and triptophenolide could be synthesized from tetracycle **3**, [28] the butenolide installation could be accomplished from key intermediate tricyclic ketone **4**,^1^ [35–38] which is accessible by a 3-step transformation including Claisen rearrangement, alkene oxidation and aldol cyclization. *Trans*-cyclization could be realized if dehydration occurs at C6-C7 after diasteroselective aldol reaction. The quaternary stereocenter in **6** could be constructed by above mentioned coupling reaction.

## Results and Discussion

**Scheme 2:**
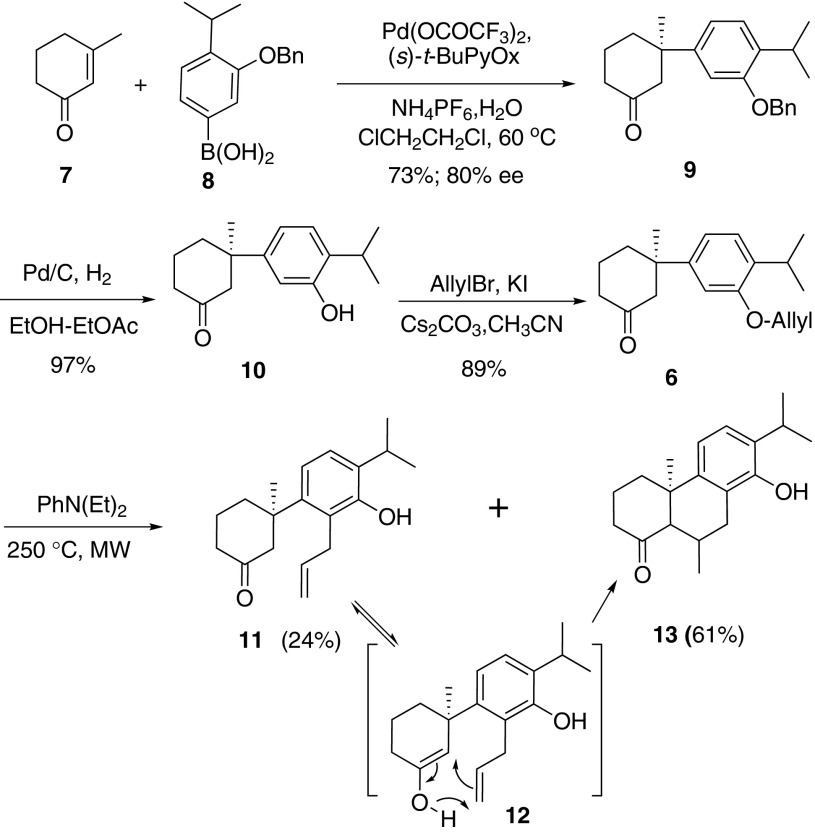
Construction of quaternary stereocenter and overreacted rearrangement

Our synthesis commenced with coupling reaction of arylboronic acid **8** [15]. Commercially available 3-Me-cyclohexenone was treated with trifluoroacetic palladium and (*s*)-*t*-BuPyOX ligand in heterogeneous solvent and **9** was isolated in moderate *ee* and 73 % yield [32]. The absolute configuration was determined accordingly [32]. Removal of Benzyl group by hydrogenation and allylation with allyl bromide afforded **6** as precursor of Claisen rearrangement. Treatment of **6** under microwave irradiation for 40 min gave rise to anticipated allyl phenol **11** as a minor product, together with tricyclic phenol **13** which was a product of Conia-ene reaction starting from **11** (Scheme 2). Unfortunately, attempts were failed to improve the yield of **11** by controlling reaction time and temperature. Alternative strategy need to be adopted to avoid side reactions.

**Scheme 3:**
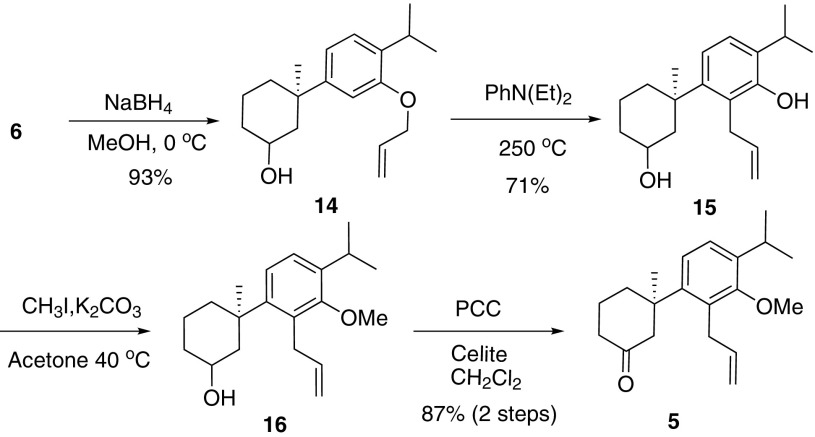
Successful claisen rearrangement

As illustrated in Scheme 2, tautomerization of ketone into enol was the reason of side reaction. Consequently, prevention of the tautomerization by reduction of ketone with NaBH_4_ could be a choice. In fact, under the same thermo condition, *ortho*-claisen rearrangement of **14** afforded diol **15** in 71 % yield (Scheme 3). Routine methyl protection of phenol and subsequent PCC oxidation produced ketone **5** in 87 % yield in 2 steps.

**Scheme 4:**
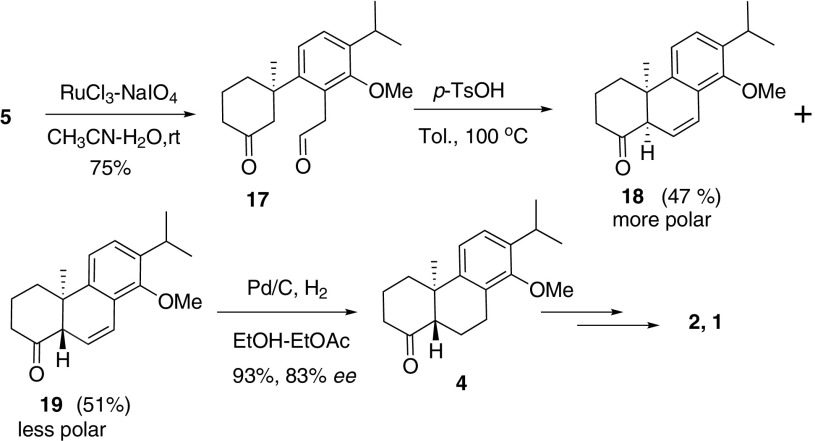
Aldol cyclization

RuCl_3_ oxidation of terminal alkene **5** to corresponding aldehyde **17** (75 % yield) set up the stage for diastereoselective aldol reaction (Scheme 4). Both isomers were isolated and *trans*-isomer was determined by NMR spectrum analysis. We were delightful that dehydration (C6-C7) proceeded in situ to give a thermodynamically stable aromatic alkene **19** in 51 % isolated yield (dehydration at C5-C6 was also possible, however, we did not detect such intermediate by TLC monitor at rt or higher temperature). TLC analysis showed that *cis*-isomer **18** diminished when reaction temperature was elevated. Hence, acceptable yield was obtained by adding *p*-TsOH at higher temperature. This provides a method to build *cis* backbone of 6-6-6 structure which is difficult to access by acidic cyclization [39]. However, after screening of different acids (Camphorsulfonic acid, TiCl_4_, BF_3_·OEt_2_, Proline) and bases (KOH, K_2_CO_3_, KO-*t*-Bu), no further improvement was realized to produce more *trans* isomer. Hydrogenation of **19** furnished key intermediate **4** in 93 % yield and the *ee* value of **4** was determined to be 83 %. The spectra of **4** were identical with reported data [28–30]. From **4**, (+)-TP and (−)-Triptophenolide could be synthesized with reported methods [28].

In summary, the first catalytic asymmetric formal total synthesis of (+)-Triptolide and (−)-Triptophenolide have been accomplished in 10 steps with 13 % yield. Key reactions involve a catalytic asymmetric construction of quaternary stereocenter and subsequent ring close via Claisen rearrangement, aldol reaction. This work demonstrated a new strategy for asymmetric construction of tricyclic ketone like **4**. Further researches on this application in total synthesis of bioactive natural products are ongoing in our lab.

## Electronic supplementary material

Below is the link to the electronic supplementary material.

Supplementary material 1 (PDF 1439 kb). Supplementary material is available in the online version of this article, which is accessible for authorized users

## Footnotes

1 See representative references for catalytic asymmetric construction of 6,6,6-tricycles.
