# Supplementary material for: Catalytic Asymmetric Formal Total Synthesis of (−)-Triptophenolide and (+)-Triptolide
Source: Nat Prod Bioprospect. 2016 Apr 20;6(3):183–6. doi: 10.1007/s13659-016-0100-z (PMC5385656; doi:10.1007/s13659-016-0100-z)
Supplement: Supplementary file 1 — Supplementary material 1 (PDF 1439 kb). Supplementary material is available in the online version of this article, which is accessible for authorized users [file 13659_2016_100_MOESM1_ESM.pdf]

Catalytic Asymmetric Formal Total Synthesis of (-)-Triptophenolide and (+)-Triptolide.

Wen-dan Xu, [‡,a,b] Liang-Qun Li, [‡,a,b] Ming-Ming Li, [c] Hui-Chun Geng, [a,b] Hong-Bo Qin*[,a]

General Experimental Procedures: All reactions were performed with dry solvents under anhydrous conditions, unless otherwise noted. Dry tetrahydrofuran (THF) were distilled over sodium. Dichloromethane were distilled over calcium hydride. Starting materials and reagents used in reactions were obtained commercially from Aladdin, Acros, Aldrich and were used without purification, unless otherwise indicated. Silica gel (200-300 mesh, Qingdao Marine Chemical Ltd., China), light petroleum ether (bp 60–90 °C) and ethyl acetate were used for product purification by flash column chromatography. Claisen rearrangement reaction was performed by CEM Discover microwave synthesizer. Proton nuclear magnetic resonance (^{1}H-NMR) spectra were recorded on Bruker Avance 400 and 500 spectrometer at 400 and 500 MHz. Carbon-13 nuclear magnetic resonance (^{13}C-NMR) was recorded at 100 and 125 MHz. IR spectra were recorded with KBr pellets on a Bruker Tensor 27 FT-IR spectrometer. Mass spectra were recorded on a VG-Auto-Spec-3000 spectrometer.

Experimental

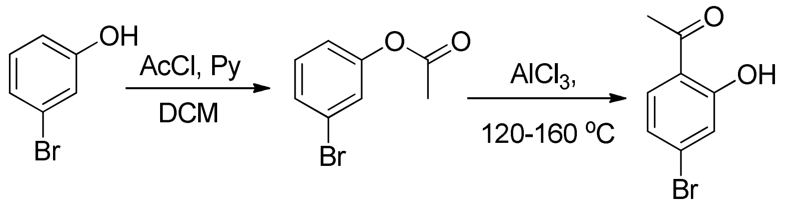

To a solution of bromophenol (20 g, 8.0 mmol) in dry CH_2Cl_2 (39 mL) was added

pyridine (12 mL) at at 0 °C, then AcCl (12 mL) was injected drop-wise slowly under argon.The mixture was stirred at room temperature for 3 h. The reaction was quenched with H_2O and extracted with CH_2Cl_2 (3×100 mL). The combined organic phases were washed with H_2O, brine, dried over Na_2SO_4 and concentrated under reduced pressure.

To a solution of the crude acetic 3-bromo benzoate (12.5 g) in dry CH_2Cl_2 (40 mL) was added $AlCl_3$ (28 g, 0.21mol) portion-wise at room temperature. The reaction mixture was stirred at 130 °C for 3 h. The resulting mixture was cooled at room temperature and dissolved with CH_2Cl_2. the reaction was quenched with H_2O, the aqueous layer was extracted with CH_2Cl_2 (3×100 mL). The combined organic phases were washed with H_2O, brine, dried over Na_2SO_4 and concentrated under reduced pressure. The product was used directly in the next step.

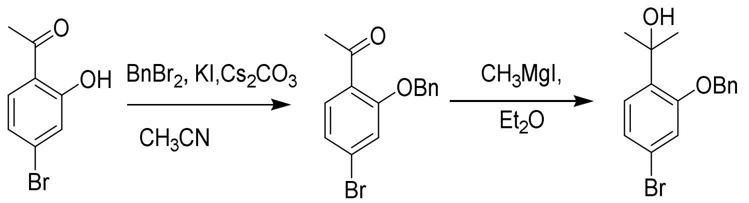

To a suspension of phenol (25.2 g, 0.1mol), anhydrous Cs_2CO_3 (47 g ,0.15mol, 1.5eq)) and KI (16.6 g, 0.1 mol, 1.0eq) in CH_3CN (100 mL) was added BnBr (12 mL, 0.1mol, 1.0eq), and the mixture was stirred at room temperature until the starting phenol was completely consumed. The mixture was filtered and the solvent was removed by rotary evaporation. The crude product was purified by flash column silica gel chromatography (PET: EA = 15:1) to give ketone (24.8 g) as faint yellow solid in 93 % yield.

Methyl iodide (0.68 mL, 78.5 mmol, 1.3 eq) in ether (10 mL) under argon was added drop-wise to a suspension of magnesium turnings (260 mg, 10.86 mmol, 1.3eq) in dry ether (10 mL). After the magnesium turnings disappeared, a solution of methyl aryl ketone (2.55 g, 8.36 mmol) in dry THF (15 mL) was added slowly at 0 °C. The reaction mixture was then allowed to warm to room temperature. The reaction completedin 3 hrs . The reaction was quenched with saturated ammonium chloride, extracted with EtOAc (3×25 mL). The combined organic layers were washed with

brine, dried over Na_2SO_4, filtered, and concentrated under reduced pressure.

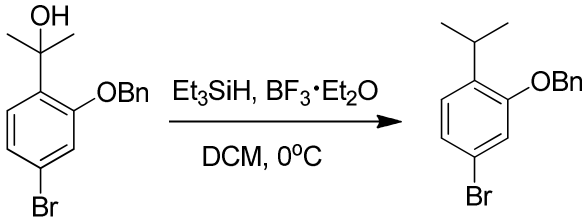

To a stirred solution of crude tertiary alcohol (5.2 g,16.2 mmol) and triethylsilane (5.18 mL, 32.5 mmol,2.0 eq) in CH_2Cl_2e (20 mL) at 0 °C was added drop-wise $BF_3 \cdot Et_2O$ (1.62 mL, 16.2mmol, 1.0 eq). The solution was allowed to warm to room temperature and stirred for 20 minutes. The reaction mixture was quenched with saturated aqueous $NaHCO_3$ (50 mL) and was extracted with CH_2Cl_2 (3×15 mL). The combined organic layers were washed with brine, dried over Na_2SO_4, filtered, and concentrated under reduced pressure. The crude product was purified by flash column silica gel chromatography (PET) to give bromobenzene (3.98 g) as colorless oil in 95% yield.

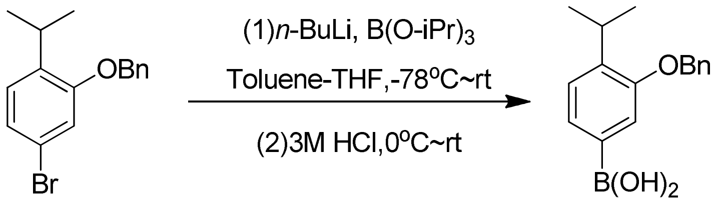

To a stirred -78 °C solution of bromobenzene (3.98g, 13.1 mmol) in Toluene/THF (V:V=2:1,30 mL) under argon was added drop-wise a solution of *n*-butyllithium (6.3 mL,15.67 mmol,1.2 eq, 2.5 M in hexane). The resulting mixture was stirred at -78 °C for 1 hour, then tri-isopropyl borate (4.56mL, 19.65 mmol,1.2 eq) was added slowly. The mixture was allowed to warm to room temperature and worked up with 3M HCl (20 mL). Afterstirring for 1 hourthe resulted mixture was extracted with EtOAc (3 × 15 mL). The combined organic layers were washed with brine, dried over Na_2SO_4, filtered, and concentrated under reduced pressure. The crude product was purified by flash column silica gel chromatography (PET: EA = 1:1) to give aryl boronic acid (2.41 g) as white solid in 68% yield.

M.P.:123-125°C. ^1H NMR (400 MHz, DMSO) δ 6.90 (m, 3H), 6.85 – 6.79 (m, 3H), 6.76 (m, 1H), 6.60 (d, *J* = 7.5 Hz, 1H), 4.53 (s, 2H), 2.76 (m, 1H), 0.59 (d, *J* = 6.9 Hz, 6H). ^{13}C NMR (100 MHz, DMSO) δ 155.28, 138.78, 138.09, 128.91, 128.12,128.12,127.75, 127.45, 127.45, 125.48,117.70, 69.7, 26.95, 22.96, 22.96. IR (neat): *v* 3440, 3431, 2961, 1606, 1503, 1455, 1408, 1342, 1268, 1232, 732,694.

HREIMS *m/z*: calcd. for $C_{16}H_{19}BO_3$ $[M]^+$: 270.1427, found: 270.1415.

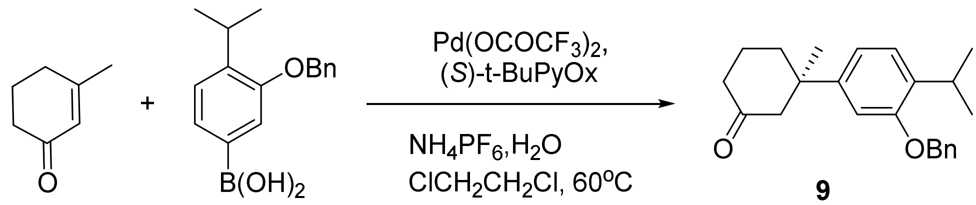

9

Synthesis of compound **8**: A screw-top dram vial was charged with a stir bar, $Pd(OCOCF_3)_2$ (12.5 mg, 0.0375 mmol, 5% eq), (*S*)-t-BuPyOX (9.2 mg, 0.045 mmol, 6% eq), NH_4PF_6(37 mg, 0.225 mmol, 0.3eq), and the aryl boronic acid **8** (405 mg, 1.5 mmol, 2.0 eq). The mixtures were suspended in CH_2Cl_2 (2 mL) and stirred for 2 min at room temperature. After a while a yellow suspension was formed. 3-methyl-2-cyclohexenone **7** (83 mg, 0.75 mmol, 1.0 eq) and water (68μL, 3.75 mmol, 5.0 eq) were added. The walls of the vial were rinsed with an additional portion of CH_2Cl_2 (0.5 mL). The mixture was stirred at 60°C in an oil bath for 24 hrs. Upon complete consumption of the starting material (monitored by TLC, 4:1 hexanes/EtOAc),the mixture was filtrated and the filtrate was further concentrated. The crude product was purified by flash column silica gel chromatography (PET: EA = 50:1) to give compound **9** (183 mg) as colorless oil in 73 % yield and 80% *ee* by chiral HLPC analysis.

^1H NMR (400 MHz, $CDCl_3$) δ 7.46 (d, *J* = 7.3 Hz, 2H), 7.38 (t, *J* = 7.4 Hz, 3H), 7.16 (d, *J* = 8.0 Hz, 1H), 6.89 – 6.84 (m, 2H), 5.08 (s, 2H), 3.35 (septet, *J* = 6.9 Hz, 1H), 2.84 (d, *J* = 14.2 Hz, 1H), 2.42 (d, *J* = 14.2 Hz, 1H), 2.29 (t, *J* = 6.8 Hz, 2H), 2.16 – 2.10 (m, 1H), 1.91 – 1.82 (m, 2H), 1.62 (m, 1H), 1.29 (s, 3H), 1.22 (d, *J* = 6.9 Hz, 6H). ^{13}C NMR (100 MHz, $CDCl_3$) δ 211.67, 155.78, 145.94, 137.53, 135.19, 128.50, 128.50,127.73, 127.27, 127.27,126.11, 118.02, 109.72, 70.03 (s, 3H), 53.27 (s, 3H),

42.84 (s, 2H), 40.85 (s, 3H), 38.12 (s, 3H), 29.95, 26.62, 22.71, 22.69,22.05. IR (neat): *v* 2960,2871,1712,1610,1503,1455,1412,1236,1163,1025,821,739,698. HREIMS *m/z*: calcd. for $C_{23}H_{28}O_2$ $[M]^+$:336.2089, found: 336.2097

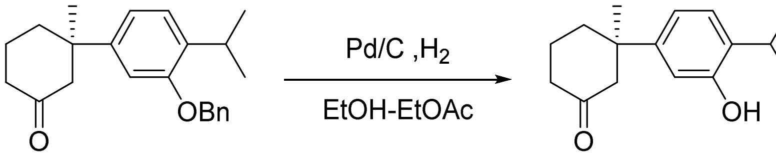

9 **10**

Ketone 9 (174 mg, 0.52 mmol) and palladium on carbon (18 mg) were added to a round bottom flask and were dried under high vacuum. Then, 1 mL of EtOAc and 1 mL of EtOH were added. The mixture were degassed twice and stirred overnight at room temperature with H_2 balloon. The crude was filtered through celite and eluted with EtOAc. The filtrate was concentrated and the crude product was purified by flash column silica gel chromatography (PET: EA = 100:1) to give phenol 10 (124 mg) as colourless oil in 97 % yield.

^{1}H NMR (400 MHz, $CDCl_3$) δ 7.12 (d, *J* = 8.1 Hz, 1H), 6.83 (d, *J* = 8.1 Hz, 1H), 6.74 (s, 1H),5.84 (br.s, 1H),3.31 – 3.08 (septet, *J* = 6.9 Hz, 1H), 2.86 (d, *J* = 14.3 Hz, 1H), 2.40 (d, *J* = 14.3 Hz, 1H), 2.31 (t, *J* = 6.9 Hz, 2H), 2.18 – 2.12 (m, 1H), 1.85 (m, 2H), 1.69 – 1.57 (m, 1H), 1.29 (s, 3H), 1.22 (d, *J* = 6.9 Hz, 6H). ^{13}C NMR (100 MHz, $CDCl_3$) δ 212.88, 152.99, 145.91, 132.24, 126.50, 117.77, 113.05, 53.10, 42.61, 40.82, 38.05,30.28,26.69,22.56,22.54,22.02.IR(neat):*v*3380,3359,2960,282,1697,1616,1580, 1418,1309,1235,1159,936,817. HREIMS *m/z*: calcd. for $C_{16}H_{22}O_2$ $[M]^+$:246.1620, found: 246.1618.

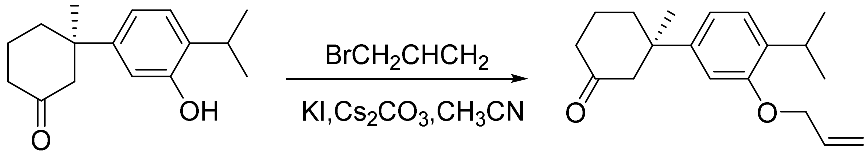

10 **6**

To a suspension of phenol **10** (249 mg, 1.0 mmol), anhydrous Cs_2CO_3 (2.02 mmol, 2.0 eq) and KI (1.01 mmol, 1.0eq) in CH_3CN (3mL) was added allyl bromide (1. 11

mol, 1.1eq), and the mixture was stirred at room temperature until the starting ketone was completely consumed. The mixture was filtered, and the solvent was removed by rotary evaporation. The crude product was purified by flash column silica gel chromatography (PET: EA = 50:3) to give compound **6** (257 mg) as colourless oil in 89 % yield.

^{1}H NMR (400 MHz, $CDCl_3$) δ 7.16 (d, *J* = 8.0 Hz, 1H), 6.87 (d, *J* = 8.0, 1H), 6.78 (s, 1H), 6.16 – 6.01 (m, 1H), 5.45 (d, *J* = 17.3, 1H), 5.28 (d, *J* = 10.6, 1H), 4.55 (d, *J* = 5.0 Hz, 2H), 3.32 (septet, *J* = 6.9 Hz, 1H), 2.86 (d, *J* = 14.2 Hz, 1H), 2.43 (d, *J* = 14.2 Hz, 1H), 2.31 (t, *J* = 6.8 Hz, 2H), 2.16 (m, 1H), 1.94 – 1.84 (m, 2H), 1.71 – 1.65 (m, 1H), 1.32 (s, 3H), 1.22 (d, *J* = 6.9 Hz, 6H).^{13}C NMR (100 MHz, $CDCl_3$) δ 211.67, 155.72, 145.89, 135.06, 133.71, 126.07, 117.89, 116.83, 109.62, 68.84, 53.27, 42.82, 40.84,38.13,29.96,26.65,22.62,22.62,22.04.IR(neat):*v*2960,2871,1713,1611,1571,1504,1413,1236,1165,1026,927,821. HREIMS *m/z*: calcd. for $C_{19}H_{26}O_2$ $[M]^+$:286.1933, found: 286.1927

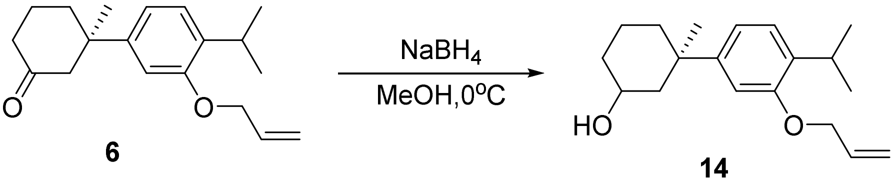

6 **14**

To a solution of carbonyl compound **6** (280 mg, 0.98 mmol) and absolute methanol (1 mL), sodium borohydride (74 mg, 1.96 mmol, 2.0eq) was added in ports at 0 ℃. The solution was then stirred at room temperature and the progress of the reaction was monitored by TLC. The reaction was quenched with saturated ammonium chloride, extracted with EtOAc (3×5 mL). The combined organic layers were washed with brine, dried over Na_2SO_4, filtered, and concentrated under reduced pressure. The crude product was purified by flash column silica gel chromatography (PET: EA = 6:1) to give corresponding alcohol **14** (256 mg) as colourless oil in 93 % yield.

^{1}H NMR (400 MHz, $CDCl_3$) δ 7.15 (d, *J* = 8.0 Hz, 1H), 6.93 (d, *J* = 8.0, 1H), 6.82 (s, 1H), 6.15 – 6.02 (m, 1H), 5.44 (d, *J* = 17.3, 1H), 5.27 (d, *J* = 10.5, 1H), 4.59 – 4.53 (m, 2H), 3.96 – 3.58 (m, 1H), 3.31 (*J* = 6.9 Hz, 1H), 2.55 (d, *J* = 12.8 Hz, 1H), 2.24 (d,

J = 12.8 Hz, 1H), 2.04 – 1.26 (m, 6H), 1.22 (d, J = 6.9 Hz, 6H), 1.18 (s, 3H).^{13}C NMR (100 MHz, $CDCl_3$) δ 155.92, 149.93, 145.38, 133.91, 125.96, 118.37, 116.76, 109.92, 68.99, 67.56, 46.99, 39.93, 36.90, 35.87, 35.26, 26.64, 22.69, 22.69, 21.06. IR (neat): *v* 3338,2934,2864,1610,1560,1502,1455,1410,1242,1106,1015,926,820.HREIMS *m/z*: calcd. for $C_{19}H_{28}O_2$ $[M]^+$:288.2089, found: 288.2090

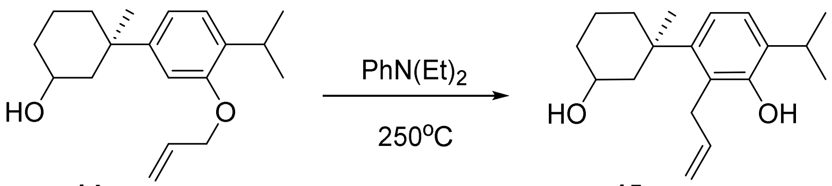

14 **15**

A mixture of compound **14** (275 mg, 4.26 mmol) and N,N-diethylaniline(1 mL) was added to a Microwave test tube (10 mL) equipped with a magnetic stirring bar and a rubber cap. The test tube was subjected to microwave reactor (CEM, Discover) at 250°C (power 300 W) for 40 minutes. After completion of the reaction, the tube was removed, cooled to room temperature, neutralized with hydrochloric acid (3 molL^{-1}) and then extracted with (3×25 mL). The combined organic layer was washed successively with water (25 mL) and brine (25 mL), dried over Na_2SO_4, filtered, and concentrated under reduced pressure. The crude product was purified by flash column silica gel chromatography (PET to give_phenol **15** (195 mg) as yellow solid in 71 % yield.

^{1}H NMR (400 MHz, $CDCl_3$) δ 7.05 (d, J = 8.3 Hz, 1H), 6.94 (d, J = 8.3 Hz, 1H), 6.26 – 5.98 (m, 1H), 5.26 (d, J = 17.3 Hz, 1H), 5.13 (d, J = 17.3 Hz, 1H), 3.81 – 3.48 (m, 3H), 3.21 (septet, J = 6.9 Hz, 1H), 2.63 (d, J = 13.1, 1H), 2.39 (d, J = 13.1 Hz, 1H), 1.95 – 1.71 (m, 2H), 1.74 – 1.52 (m, 3H), 1.33 (s, 3H), 1.18 (d, J = 6.9 Hz, 6H).^{13}C NMR (100 MHz, $CDCl_3$) δ 153.36, 142.28, 136.22, 133.32, 124.87, 124.01, 120.68, 116.94, 67.88, 48.68, 41.37, 38.85, 35.98, 33.01, , 32.25, 27.05, 22.69, 22.63, 21.56. IR(neat):*v*3537,3359,2957,2866,1555,1407,1295,1239,1192,1099,1056,1013,939. HREIMS *m/z*: calcd. for $C_{19}H_{28}O_2$ $[M]^+$:288.2089, found: 288.2082

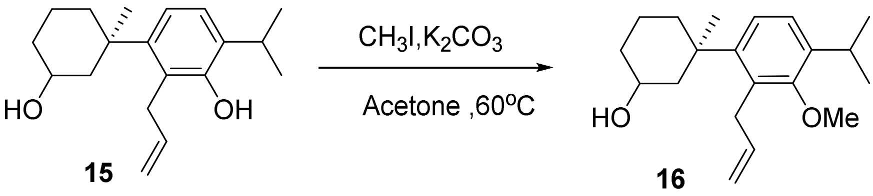

Compound **15** (195 mg, 0.68 mmol,1.0eq) was dissolved in dry Me_2CO (2.0 mL), and to the solution was added powdered potassium carbonate (169 mg, 1.22 mmol,1.8eq) and methyl iodide(63 μL, 1.01 mmol,1.5eq). The reaction mixture was stirred at 40°C for 7 h under Ar atmosphere and light-protecting condition. The solvent was evaporated under reduced pressure. Ice cooled H_2O (10 mL) was added to the residue and the aqueous phase was extracted with EtOAc (20 mL×3). The combined organic layers were washed with brine, dried over Na_2SO_4, filtered, and concentrated under reduced pressure afforded the corresponding compound **16** (203 mg), which was immediately used in the next step without further purification.

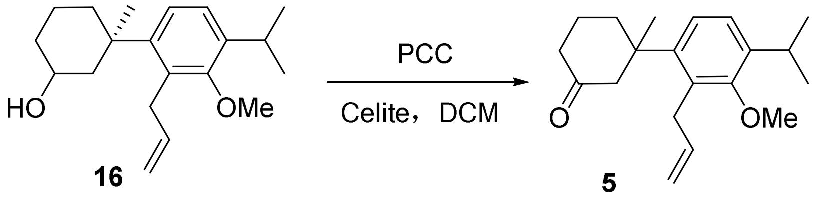

PCC（288 mg, 1.34 mmol, 2.0eq）and Celite (600 mg) were added to a solution of **16** (203 mg, mmol) in CH_2Cl_2 (2 mL).The mixture were stirred at room temperature overnight and then filtered through silica gel. pad. Removal of the solvent under reduced pressure to give crude product. It was then purified by flash column silica gel chromatography (PET: EA = 50:1) to give ketone **5** (178 mg) as colourless oil in 87% yield in 2 steps.

^{1}H NMR (400 MHz, $CDCl_3$) δ^{1}H NMR (400 MHz, $CDCl_3$) δ 7.08 (d, *J* =8.5 Hz, 1H), 7.04 (d, *J* = 8.5 Hz, 1H), 5.99 (m, 1H), 5.02 (d, *J* = 9.9 Hz, 1H), 4.80 (d, *J* = 9.9 Hz, 1H), 3.71 (s, 3H), 3.65 (s, 2H), 3.32 – 3.21 (m, 1H), 2.94 (d, *J* = 14.3 Hz, 1H), 2.41 (d, *J* = 12.0 Hz, 2H), 2.29 (s, 2H), 1.94 – 1.76 (m, 3H), 1.41 (s, 3H), 1.21 (d, *J* = 6.9 Hz, 6H).^{13}C NMR (100 MHz, $CDCl_3$) δ 211.90, 157.76, 143.33, 140.19, 137.95, 130.94, 124.80, 123.35, 114.78, 61.69, 55.93, 44.18, 40.84, 36.98, 32.54, 28.66, 26.33, 24.00,

23.82,22.10. IR (neat):v 2962,2872,1712,1457,1400,1317,1288,1228,1029,910,823. HREIMS m/z: calcd. for $C_{20}H_{28}O_2$ $[M]^+$:300.2089, found:300.2081

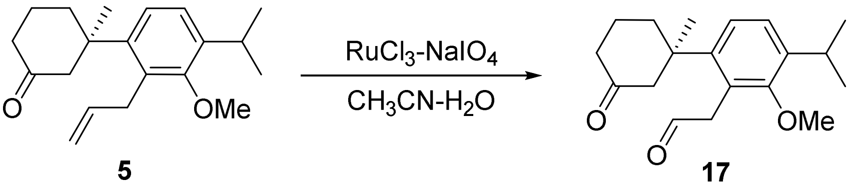

To a stirred mixture of olefin **5** (117 mg, 0.39 mmol) and $RuCl_3$ (3mg, 3.5 mol %) in CH_3CN (1.2 mL) and distilled water (0.2 mL) was added in portions NaIO4 (164 mg, 0.765 mmmol,2.0eq) over a period of 5 min at room temperature. The color turned from black to yellow immediately. The reaction was monitored by TLC. After completion in 0.5 h, the reaction was quenched with saturated aqueous solution of $Na_2S_2O_3$, extracted with EtOAc (3×10 mL). The combined organic layers were washed with water and brine, dried over Na_2SO_4, filtered, and concentrated under reduced pressure. The crude product was purified by flash column silica gel chromatography (PET: EA = 10:1) to give desired aldehyde **17** (88 mg) as colourless oil in 75% yield.

^{1}H NMR (400 MHz, $CDCl_3$) δ 9.58 (s, 1H), 7.15 (d, J = 8.4 Hz, 1H), 7.07 (d, J = 8.4 Hz, 1H), 3.83 (s, 2H), 3.62 (s, 3H), 3.24 (septet, J = 6.9 Hz, 1H), 2.93 (d, J = 14.4 Hz, 1H), 2.41 (d, J = 14.4 Hz, 1H), 2.29 (t, J = 6.9 Hz, 2H), 1.95 – 1.81 (m, 2H), 1.75 – 1.42 (m, 2H), 1.38 (s, 3H), 1.21 (d, J = 6.9, Hz, 6H). ^{13}C NMR (100 MHz, $CDCl_3$) δ 211.19, 199.59, 157.38, 143.93, 140.23, 126.09, 124.89, 123.80, 60.10, 55.68, 43.81, 43.74, 40.66, 37.10, 28.30, 26.37, 23.80, 23.75,21.75. IR (neat): v 2959, 2873, 1965, 1714, 1613, 1570, 1461, 1407, 1252,1036, 953, 821. HREIMS m/z: calcd. for $C_{19}H_{26}O_3$ $[M]^+$:302.1882, found:302.1876

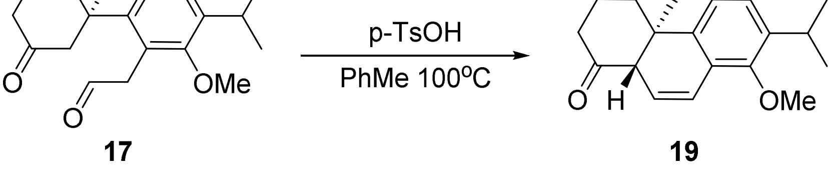

Compound **17** (45 mg, 0.15 mmol) was dissolved in dry Toluene (2.0 mL), the solution was stirred at 100°C and then added catalytic amount of p-TsOH (5 mg), the

progress of the reaction was monitored by TLC. The reaction was quenched with H_2O, extracted with EtOAc (3×5 mL). The combined organic layers were washed with brine, dried over Na_2SO_4, filtered, and concentrated under reduced pressure. The crude product was purified by flash column silica gel chromatography (PET: EA = 20:1) to give corresponding **19** (22 mg) as colourless oil in 51 % yield. ^{1}H NMR (400 MHz, $CDCl_3$) δ 7.14 (d, J = 8.1 Hz, 1H), 7.05 (d, J = 8.1 Hz, 1H), 6.85 (dd, J = 9.9, 2.8 Hz, 1H), 6.44 (dd, J = 9.9, 2.3 Hz, 1H), 3.72 (s, 3H), 3.41 (s, 1H), 3.31 (septet, J = 6.9 Hz, 1H), 2.23 (m, 6H), 1.23 (d, J = 6.8Hz, 6H), 0.97 (s, 3H). ^{13}C NMR (101 MHz, $CDCl_3$) δ 209.60, 153.92, 143.41, 139.92, 125.76, 125.63,123.76, 122.36, 118.73, 62.39, 55.06, 42.65, 40.73, 34.53, 26.22, 23.80, 23.76, 22.71, 20.16. IR (neat): v 2958, 1716, 1668, 1569, 1583, 1547, 1463, 1327, 1139, 1311, 1053,903.
HREIMS *m/z*: calcd. for $C_{19}H_{24}O_2$ $[M]^+$:284.1776, found: 284.1779

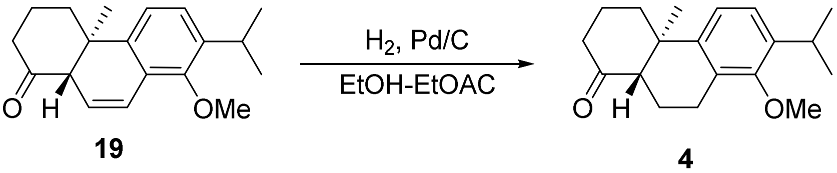

19 (15 mg, 0.05 mmol) and palladium on carbon (3 mg) were added to a round bottom flask and were dried under high vacuum. Then, 1 mL of EtOAc and 1 mL of EtOH were added. The resulting mixture were equipped with a H2 balloon and purged twice. The mixture was stirred overnight at room temperature. The crude was filtered off over celite and eluted with EtOAc. The filtrate was concentrated. The crude product was purified by flash column silica gel chromatography (PET: EA = 100:1) to give product **4** (11 mg) as colourless amorpphis in 93 % yield and 83% *ee* by chiral HLPC.

^{1}H NMR (400 MHz, $CDCl_3$) δ 7.08 (d, J = 3.9 Hz, 2H), 3.72 (s, 3H), 3.29 (septet , 6.8 Hz, 1H), 3.06 (dd, J = 17.7, 6.0 Hz, 1H), 2.67 – 2.35 (m, 5H), 2.12 – 1.72 (m, 5H), 1.56 (s, 3H), 1.22 (dd, J = 6.7, 3.4 Hz, 6H), 1.05 (s, 3H). ^{13}C NMR (100 MHz, $CDCl_3$) δ 212.54, 155.12, 144.65, 138.93, 128.52,124.04, 121.03, 60.54, 55.10, 42.42, 40.88, 37.20, 26.14, 23.92, 23.87, 23.66, 23.10, 22.59, 17.05. IR (neat): v 2960, 1714, 1478,

1437, 1424, 1307, 1300, 1291, 1116, 1054, 1032, 1008, 948, 816. HREIMS *m/z*: calcd. for $C_{19}H_{26}O_2$ $[M]^+$:, found: 286.1936

NOTE: Spectra of *cis*-isomer of **4** were loaded for the reason that after established the structure *cis*-isomer of **4** by HMQC and ROESY 2D NMR analysis, it will indirectly prove the correct assignment of **18** and *trans* isomer of **4**.

Spectra for compounds

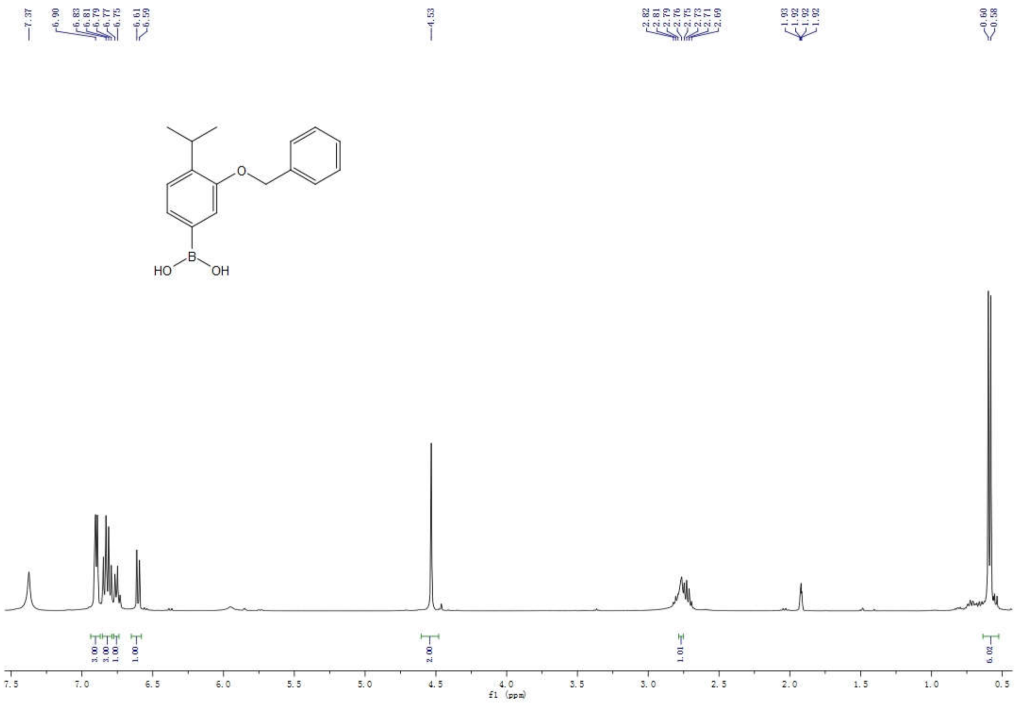

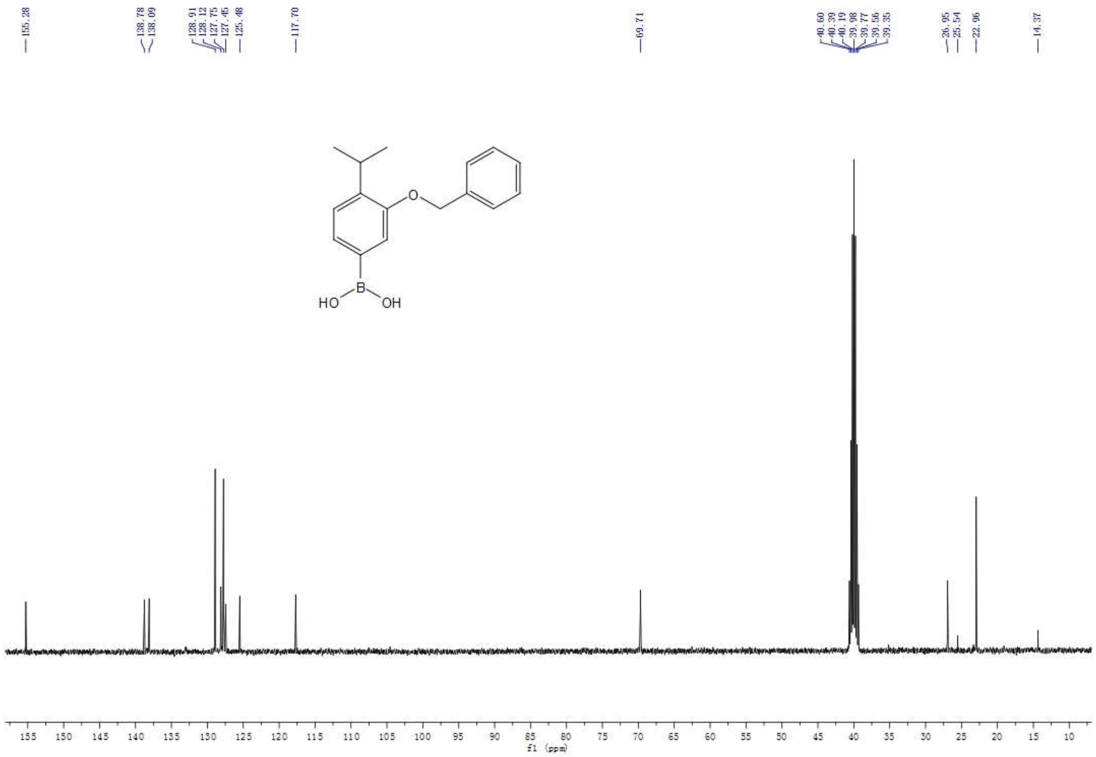

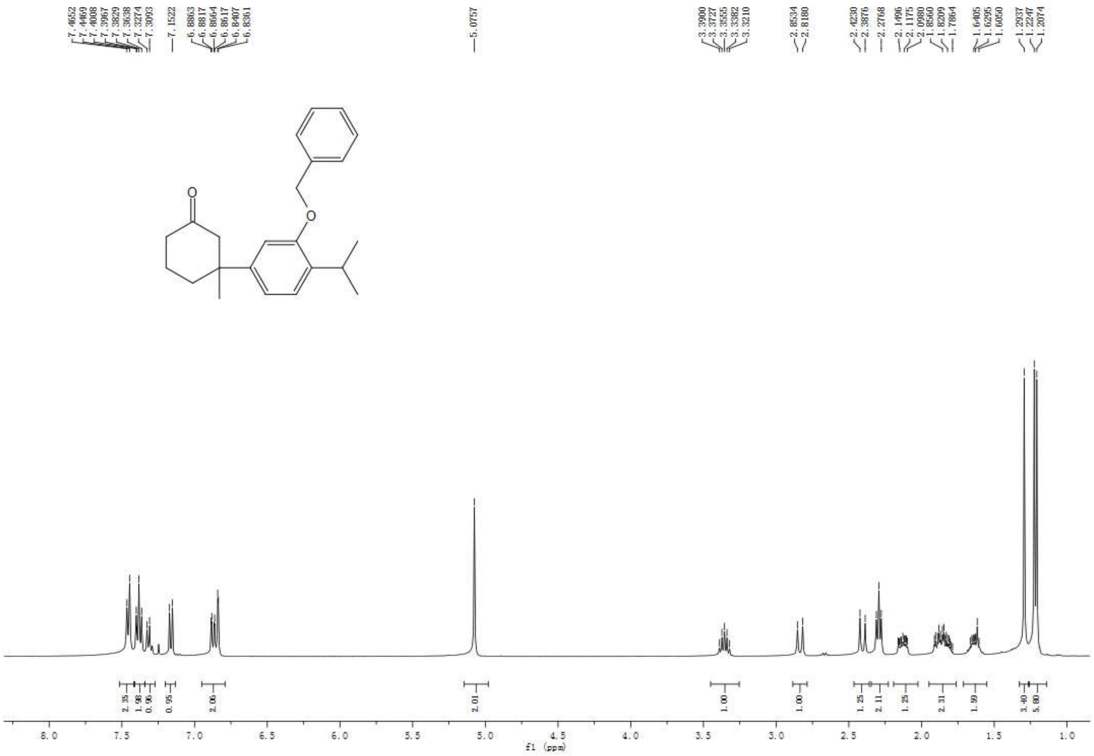
7.4652
7.4469
7.4008
7.3967
7.3829
7.3638
7.3274
7.3093
7.1522
6.8863
6.8817
6.8664
6.8617
6.8407
6.8361
5.0757
3.3900
3.3727
3.3555
3.3382
3.3210
2.8534
2.8180
2.4230
2.3876
2.2768
2.1496
2.1175
2.0980
1.8560
1.8209
1.7864
1.6405
1.6295
1.6050
1.2937
1.2247
1.2074
O
O
2.35
1.98
1.00
0.96
0.95
2.06
2.01
1.00
1.00
1.25
2.11
1.25
2.31
1.59
3.40
5.80
8.0
7.5
7.0
6.5
6.0
5.5
5.0
4.5
4.0
3.5
3.0
2.5
2.0
1.5
1.0
f1 (ppm)

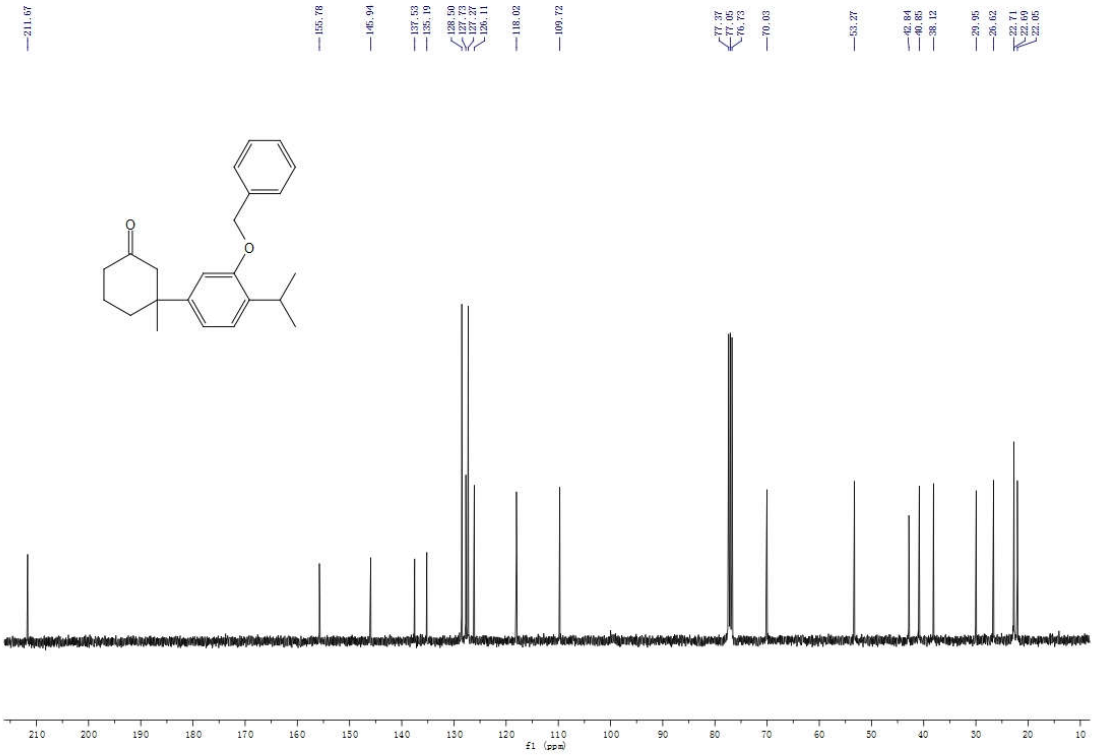
211.67
155.78
145.94
137.53
135.19
128.50
127.73
127.27
126.11
118.02
109.72
77.37
77.05
76.73
70.03
53.27
42.84
40.85
38.12
29.95
26.62
22.71
22.69
22.05
O
O
210
200
190
180
170
160
150
140
130
120
110
100
90
80
70
60
50
40
30
20
10
f1 (ppm)

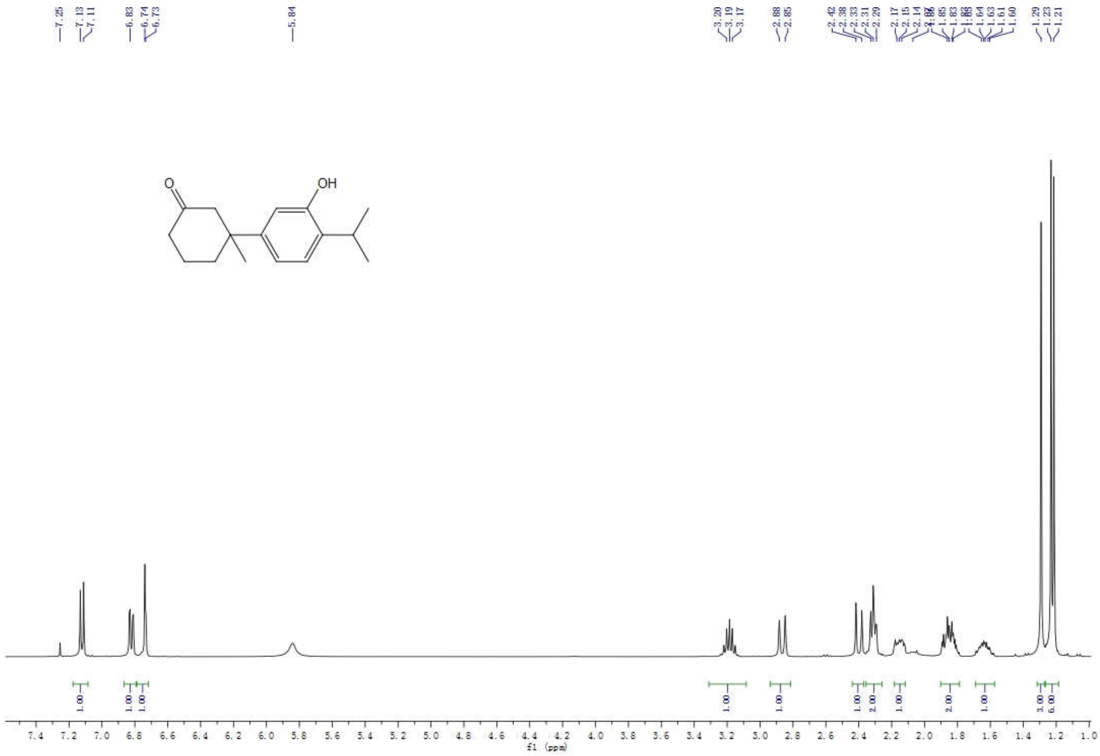
7.25
7.13
7.11
6.83
6.74
6.73
5.84
3.20
3.19
3.17
2.88
2.85
2.42
2.38
2.33
2.31
2.29
2.17
2.15
2.14
1.85
1.83
1.64
1.63
1.61
1.60
1.29
1.23
1.21
O
OH
1.00
1.00
1.00
1.00
1.00
1.00
2.00
1.00
2.00
1.00
3.00
6.00
f1 (ppm)

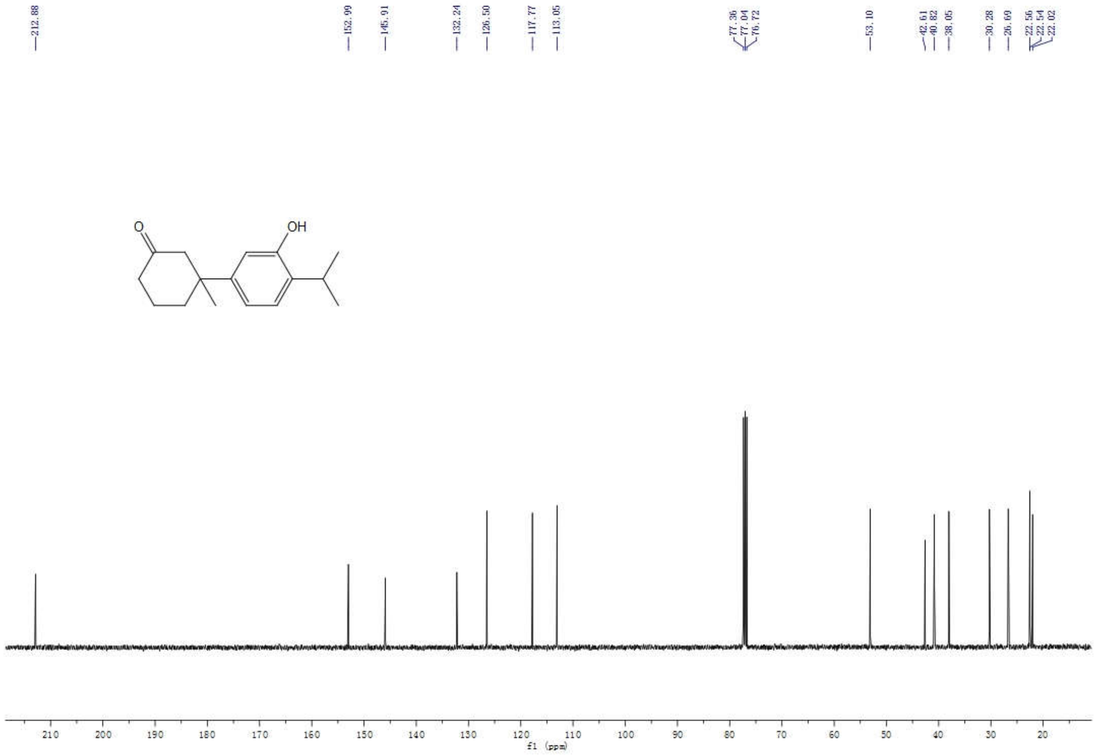
212.88
152.99
145.91
132.24
126.50
117.77
113.05
77.36
77.04
76.72
53.10
42.61
40.82
38.05
30.28
26.69
22.56
22.54
22.02
O
OH
f1 (ppm)

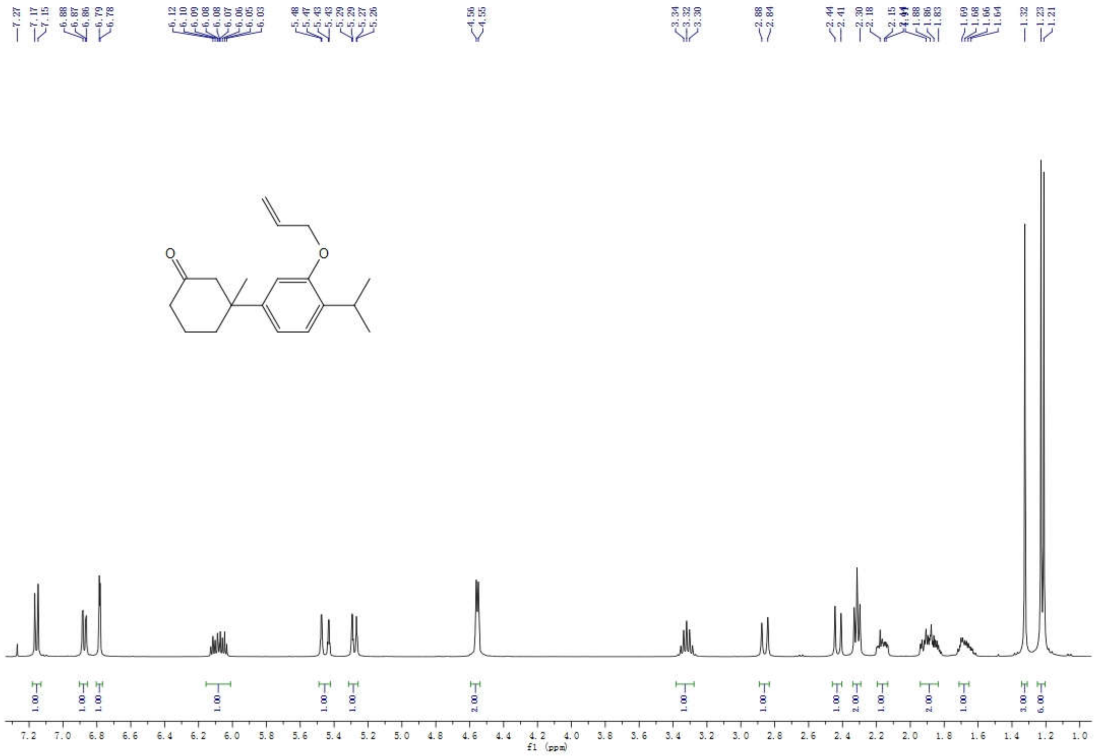
7.27 7.17 7.15 6.88 6.87 6.86 6.79 6.78
6.12 6.10 6.09 6.08 6.08 6.07 6.06 6.05 6.03
5.48 5.47 5.43 5.43 5.29 5.29 5.27 5.26
4.56 4.55
3.34 3.32 3.30
2.88 2.84
2.44 2.41 2.30 2.18 2.15
1.88 1.86 1.83
1.69 1.68 1.66 1.64
1.32 1.23 1.21
O
O
1.00 1.00 1.00 1.00 1.00 1.00 2.00 1.00 1.00 1.00 2.00 1.00 2.00 1.00 3.00 6.00
f1 (ppm)

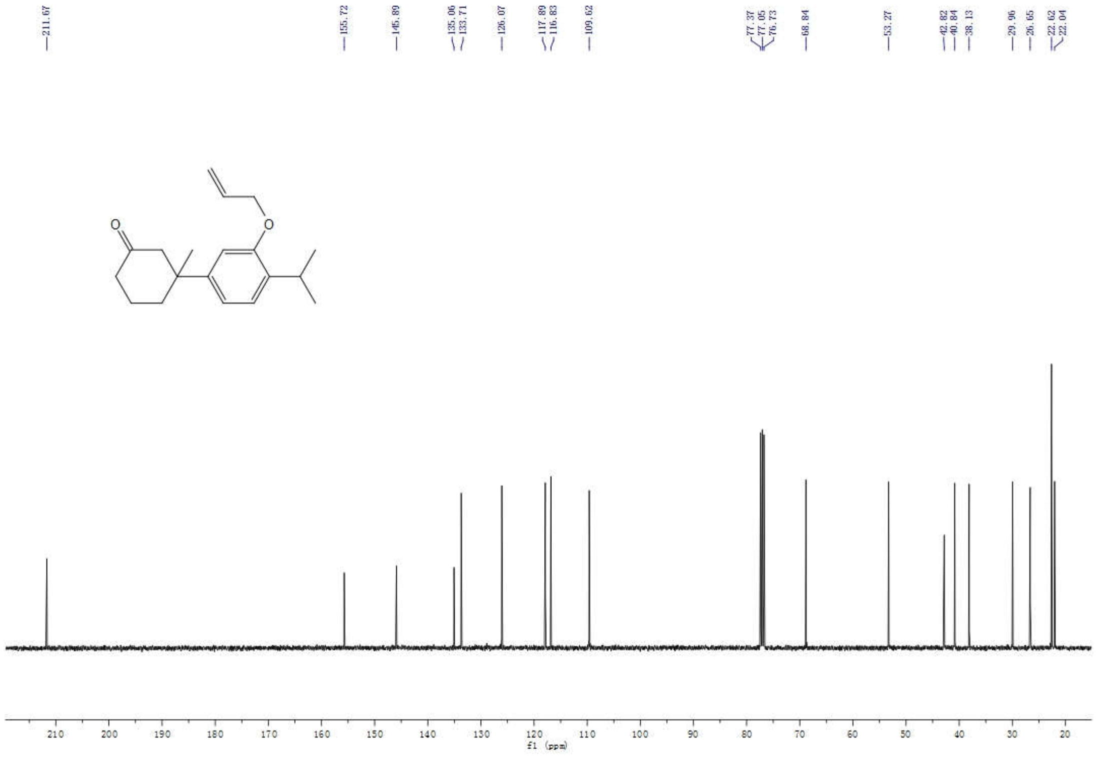
211.67
155.72 145.89 135.06 133.71 126.07 117.89 116.83 109.62
77.37 77.05 76.73 68.84 53.27 42.82 40.84 38.13 29.96 26.65 22.62 22.04
O
O
f1 (ppm)

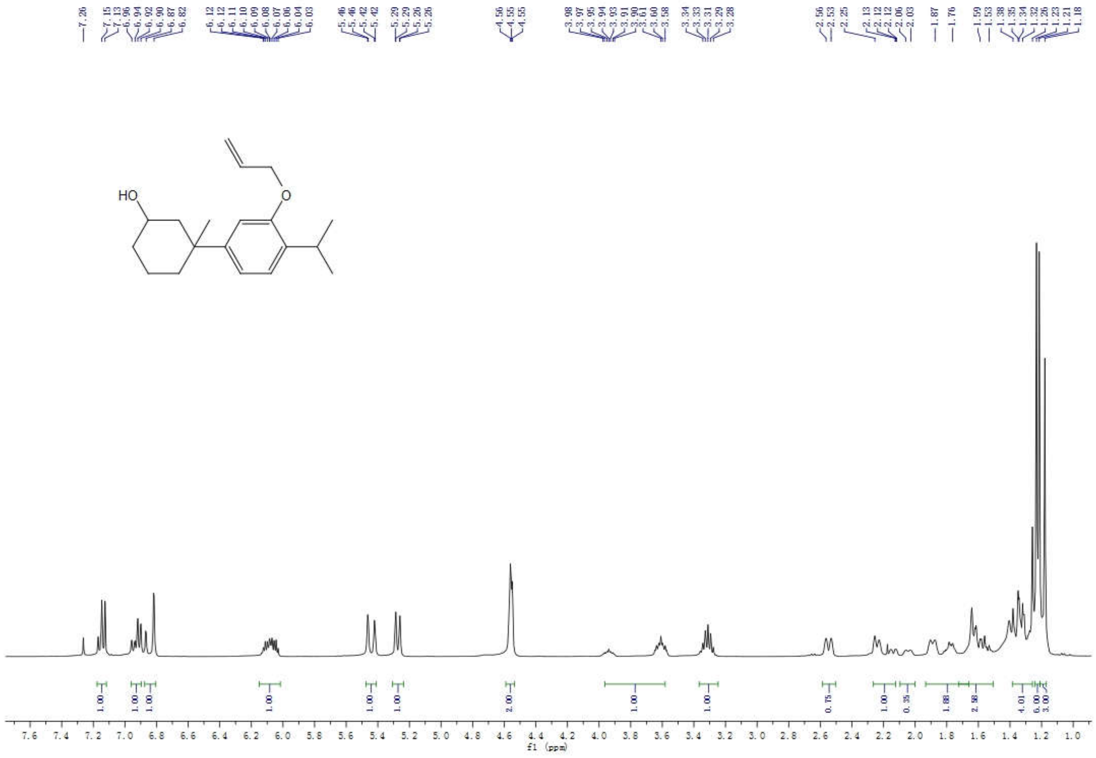
7.26 7.15 7.13 6.96 6.94 6.92 6.90 6.87 6.82
6.12 6.12 6.11 6.10 6.09 6.08 6.07 6.06 6.04 6.03
5.46 5.46 5.42 5.42 5.29 5.29 5.26 5.26
4.56 4.55 4.55
3.98 3.97 3.95 3.94 3.93 3.91 3.90 3.61 3.60 3.58 3.34 3.33 3.31 3.29 3.28
2.56 2.53 2.25 2.13 2.12 2.12 2.06 2.03 1.87 1.76
1.59 1.53 1.38 1.35 1.34 1.32 1.26 1.23 1.21 1.18
HO
O
1.00 1.00 1.00 1.00 1.00 1.00 2.00 1.00 1.00 0.75 1.00 0.35 1.88 2.58 4.01 6.00 3.00
f1 (ppm)

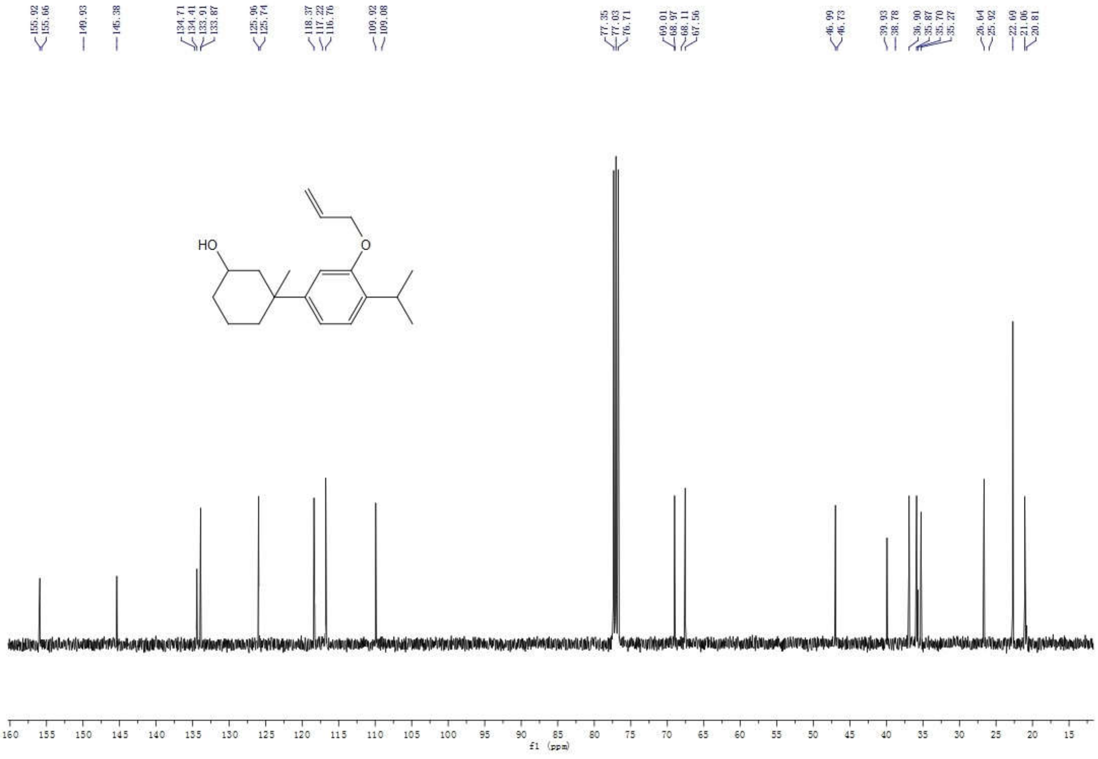
155.92 155.66 149.93 145.38 134.71 134.41 133.91 133.87 125.96 125.74 118.37 117.22 116.76 109.92 109.08
77.35 77.03 76.71 69.01 68.97 68.11 67.56
46.99 46.73 39.93 38.78 36.90 35.87 35.70 35.27 26.64 25.92 22.69 21.06 20.81
HO
O
f1 (ppm)

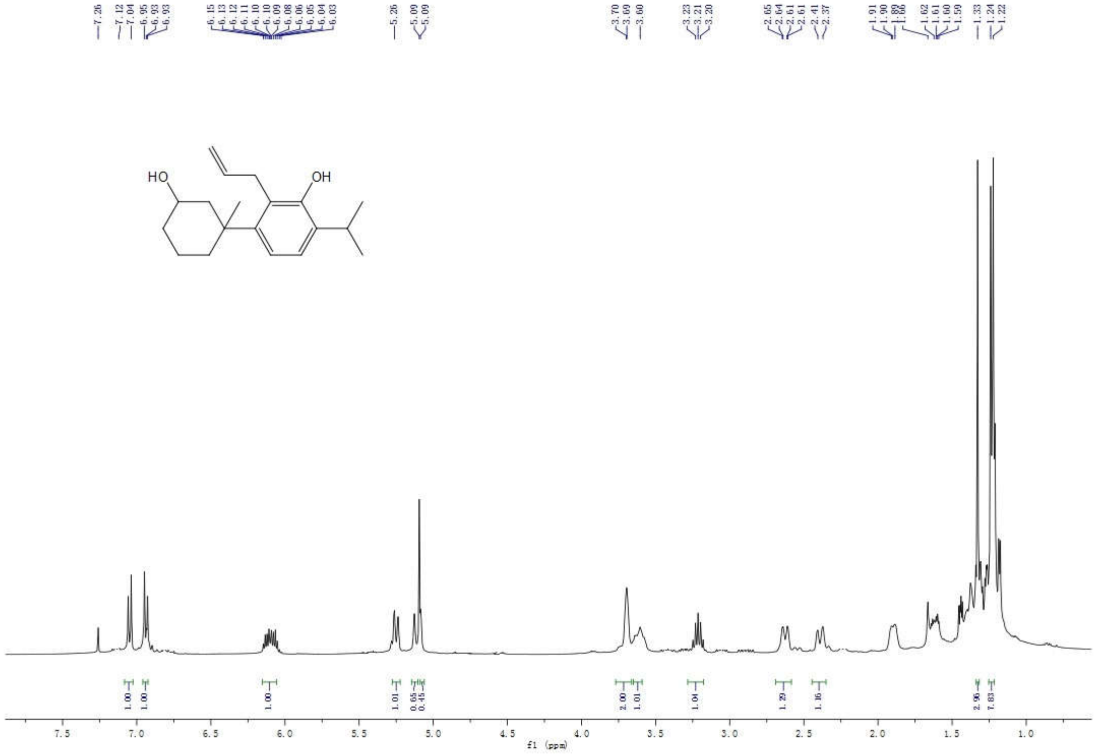
7.26
7.12
7.04
6.95
6.93
6.93
6.15
6.13
6.12
6.11
6.10
6.10
6.09
6.08
6.06
6.05
6.04
6.03
5.26
5.09
5.09
3.70
3.69
3.60
3.23
3.21
3.20
2.65
2.64
2.61
2.61
2.41
2.37
1.91
1.90
1.62
1.61
1.60
1.59
1.33
1.24
1.22
HO
OH
f1 (ppm)

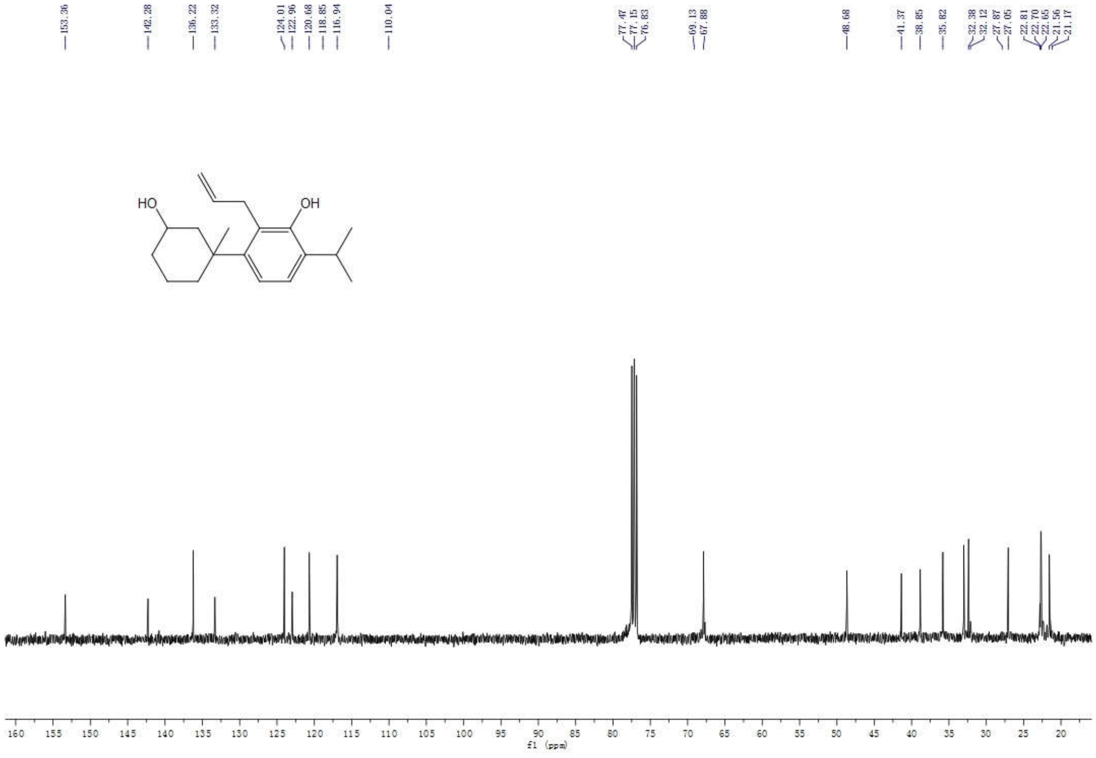
153.36
142.28
136.22
133.32
124.01
122.96
120.68
118.85
116.94
110.04
77.47
77.15
76.83
69.13
67.88
48.68
41.37
38.85
35.82
32.38
32.12
27.87
27.05
22.81
22.70
22.65
21.56
21.17
HO
OH
f1 (ppm)

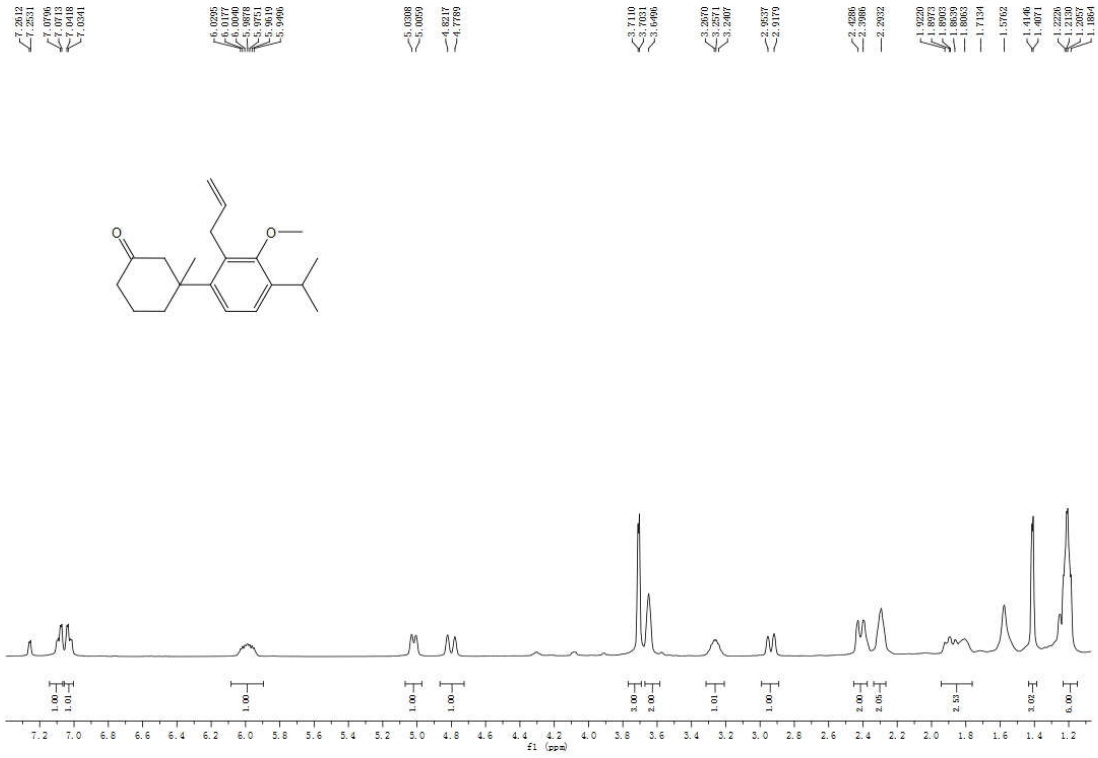
7.2612
7.2531
7.0796
7.0713
7.0418
7.0341
6.0295
6.0177
6.0040
5.9878
5.9751
5.9619
5.9496
5.0308
5.0059
4.8217
4.7789
3.7110
3.7031
3.6496
3.2670
3.2571
3.2407
2.9537
2.9179
2.4286
2.3986
2.2932
1.9220
1.8973
1.8903
1.8639
1.8063
1.7134
1.5762
1.4146
1.4071
1.2226
1.2130
1.2057
1.1864
O
1.00
1.01
1.00
1.00
1.00
3.00
2.00
1.01
1.00
2.00
2.05
2.53
3.02
6.00
f1 (ppm)

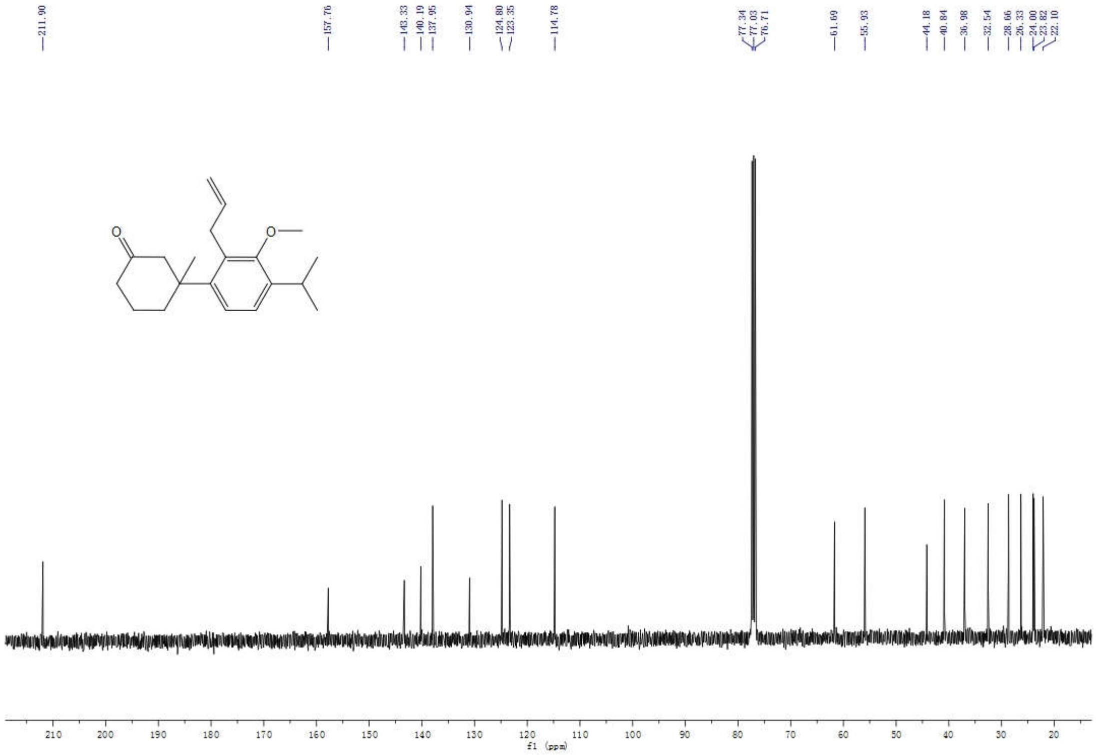
211.90
157.76
143.33
140.19
137.95
130.94
124.80
123.35
114.78
77.34
77.03
76.71
61.69
55.93
44.18
40.84
36.98
32.54
28.66
26.33
24.00
23.82
22.10
O
f1 (ppm)

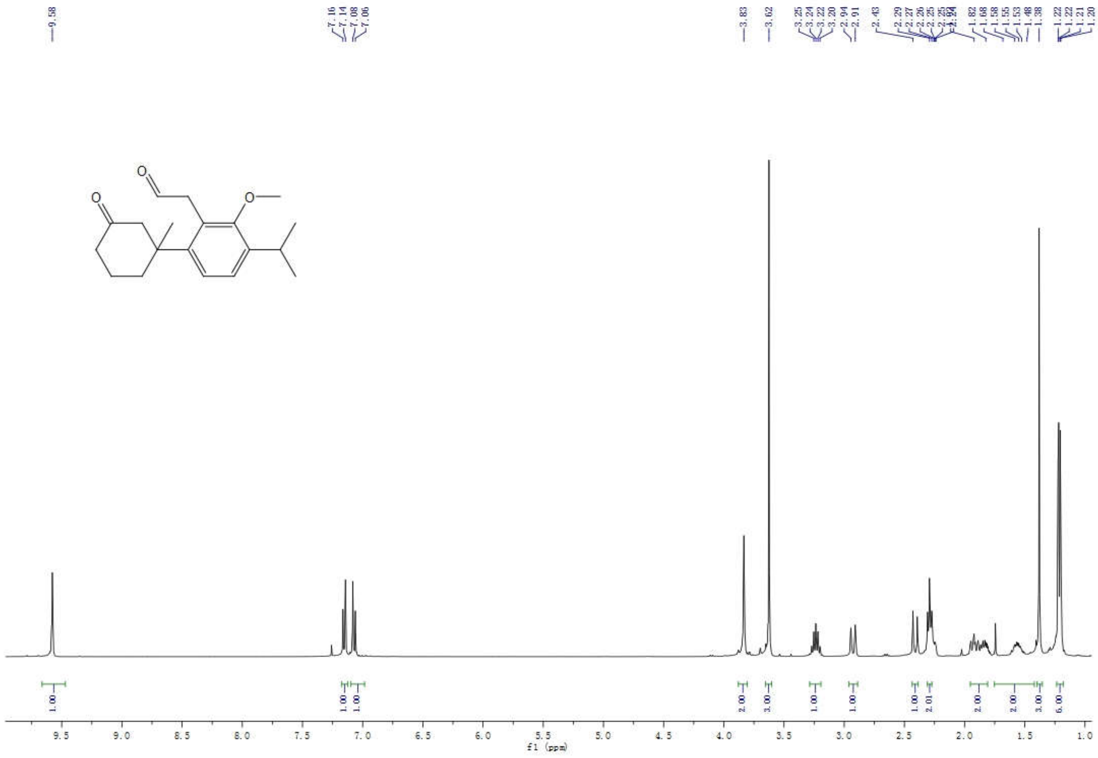
9.58
7.16
7.14
7.08
7.06
3.83
3.62
3.25
3.24
3.22
3.20
2.94
2.91
2.43
2.29
2.27
2.26
2.25
1.82
1.68
1.58
1.55
1.53
1.48
1.38
1.22
1.22
1.21
1.20
O
O
O
1.00
1.00
1.00
2.00
3.00
1.00
1.00
1.00
2.01
2.00
2.00
3.00
6.00
f1 (ppm)

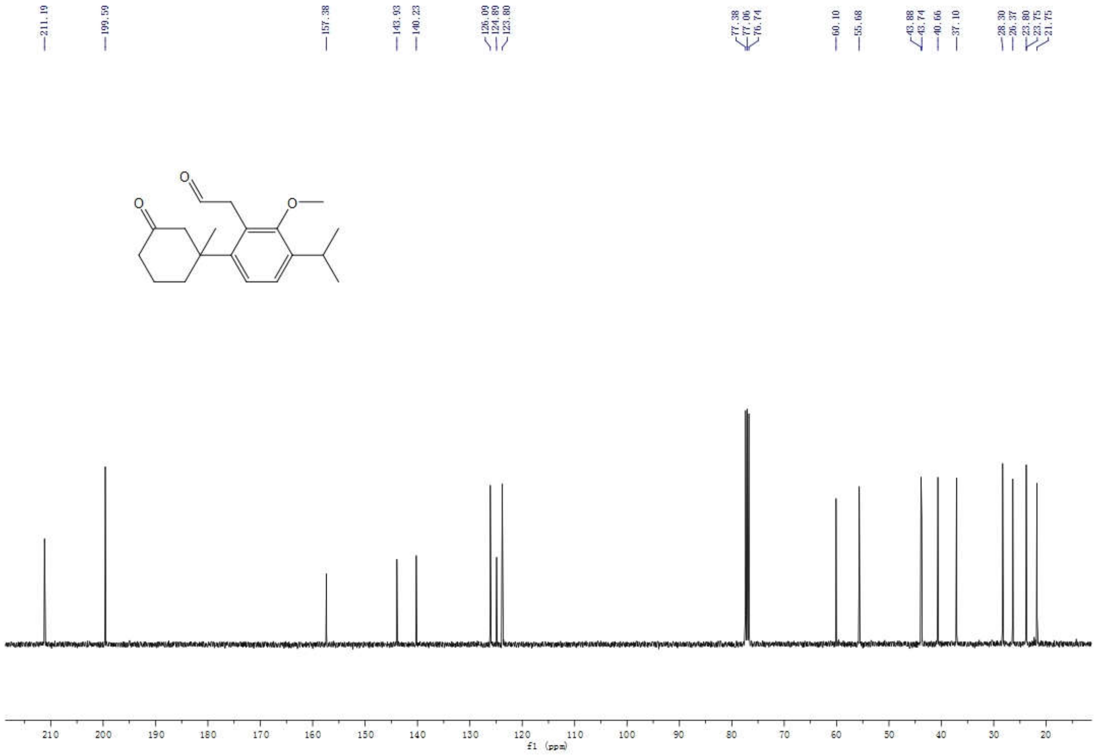
211.19
199.59
157.38
143.93
140.23
126.09
124.89
123.80
77.38
77.06
76.74
60.10
55.68
43.88
43.74
40.66
37.10
28.30
26.37
23.80
23.75
21.75
O
O
O
f1 (ppm)

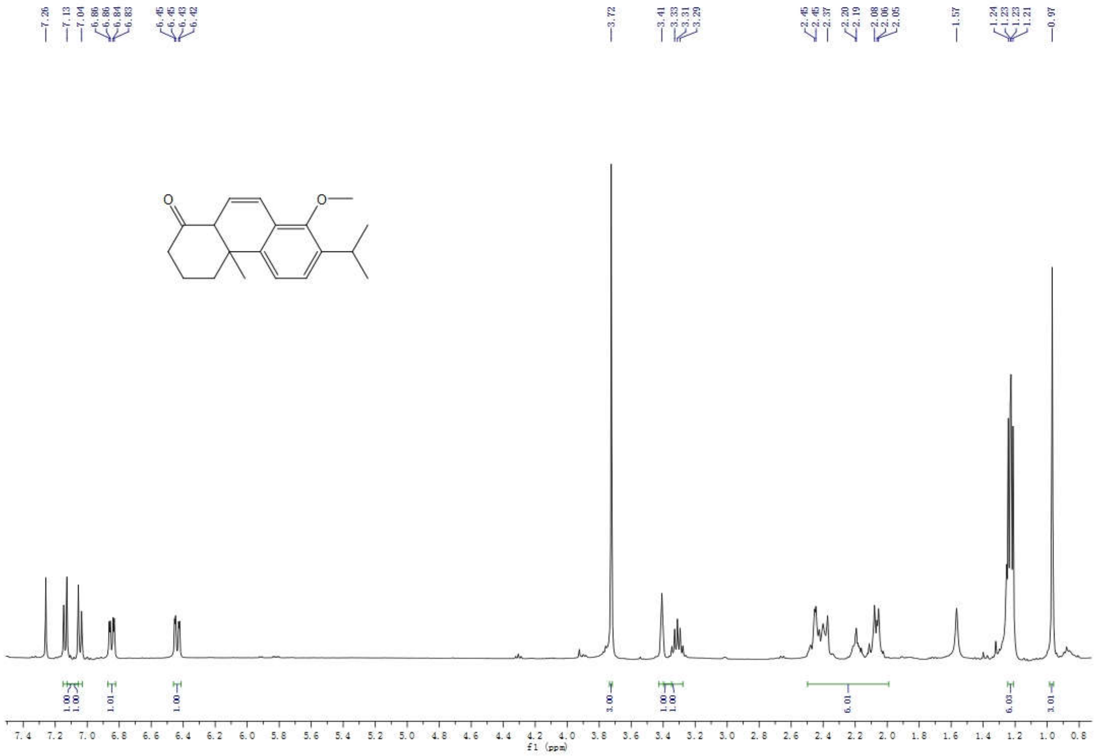
7.26
7.13
7.04
6.86
6.86
6.84
6.83
6.45
6.45
6.43
6.42
3.72
3.41
3.33
3.31
3.29
2.45
2.45
2.37
2.20
2.19
2.08
2.06
2.05
1.57
1.24
1.23
1.23
1.21
0.97
O
1.00
1.00
1.01
1.00
3.00
1.00
1.00
6.01
6.03
3.01
f1 (ppm)

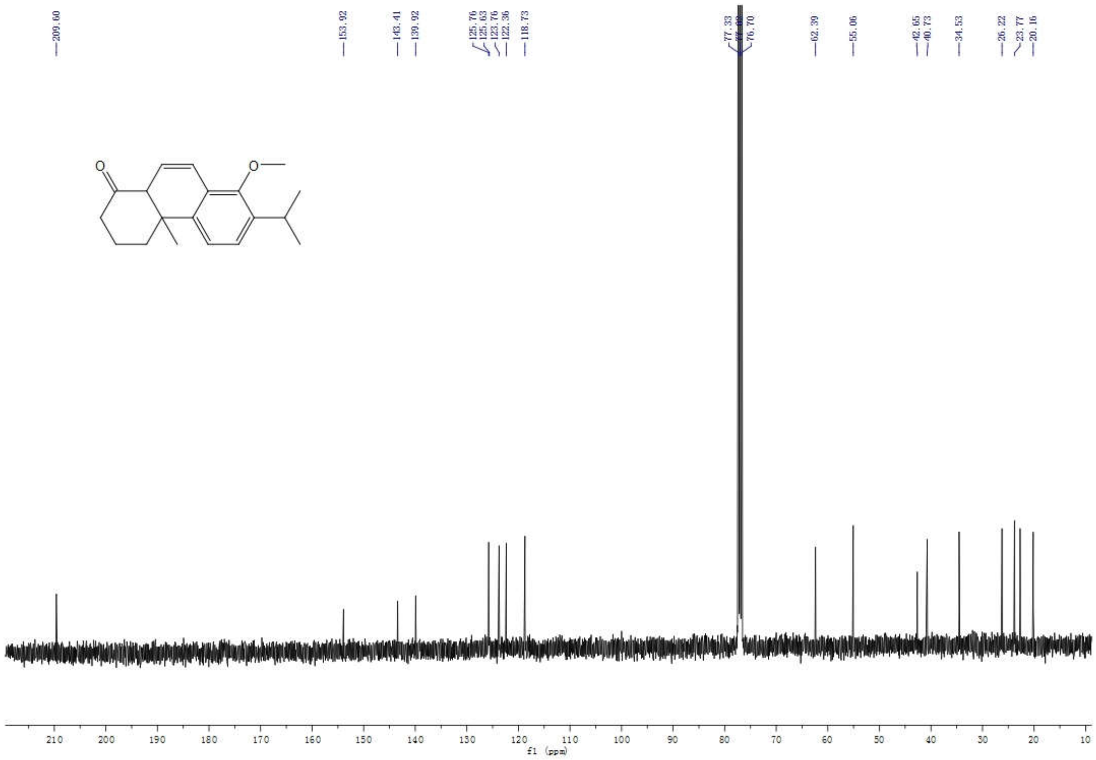
209.60
153.92
143.41
139.92
125.76
125.63
123.76
122.36
118.73
77.33
76.70
62.39
55.06
42.65
40.73
34.53
26.22
23.77
20.16
O
f1 (ppm)

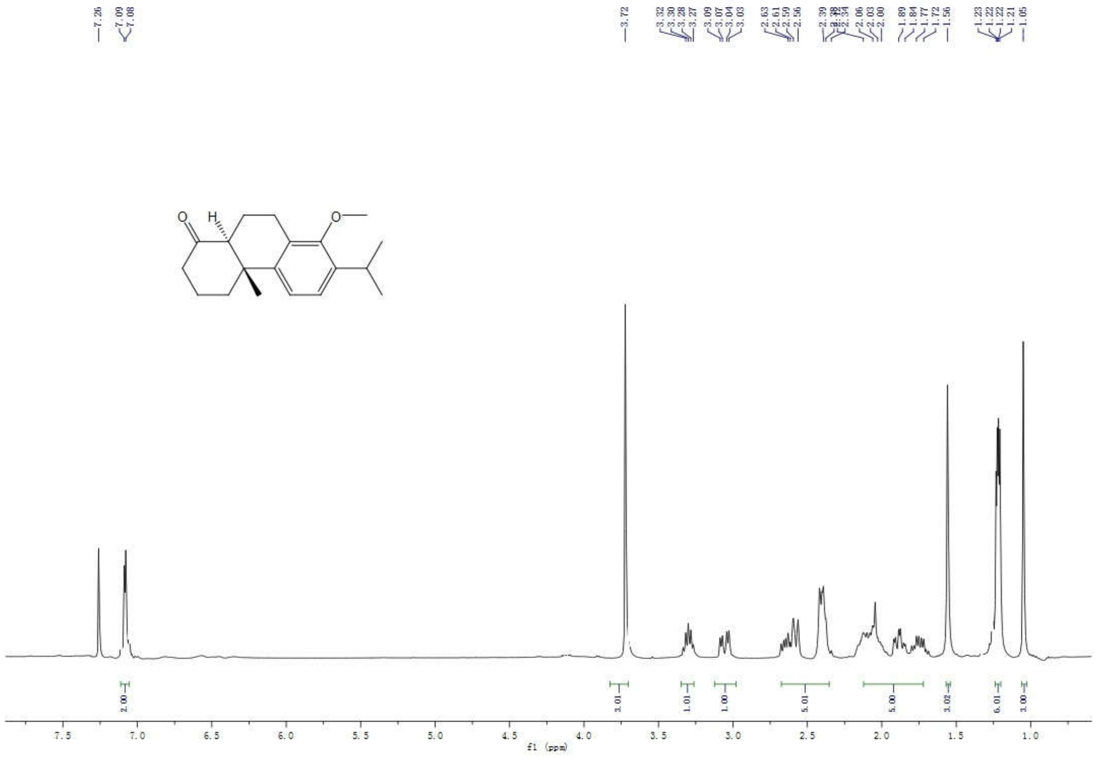

7.26
7.09
7.08
3.72
3.32
3.30
3.28
3.27
3.09
3.07
3.04
3.03
2.63
2.61
2.59
2.56
2.39
2.34
2.06
2.03
2.00
1.89
1.84
1.77
1.72
1.56
1.23
1.22
1.22
1.21
1.05
O
H
O
2.00
3.01
1.01
1.00
5.01
5.00
3.02
6.01
3.00
f1 (ppm)

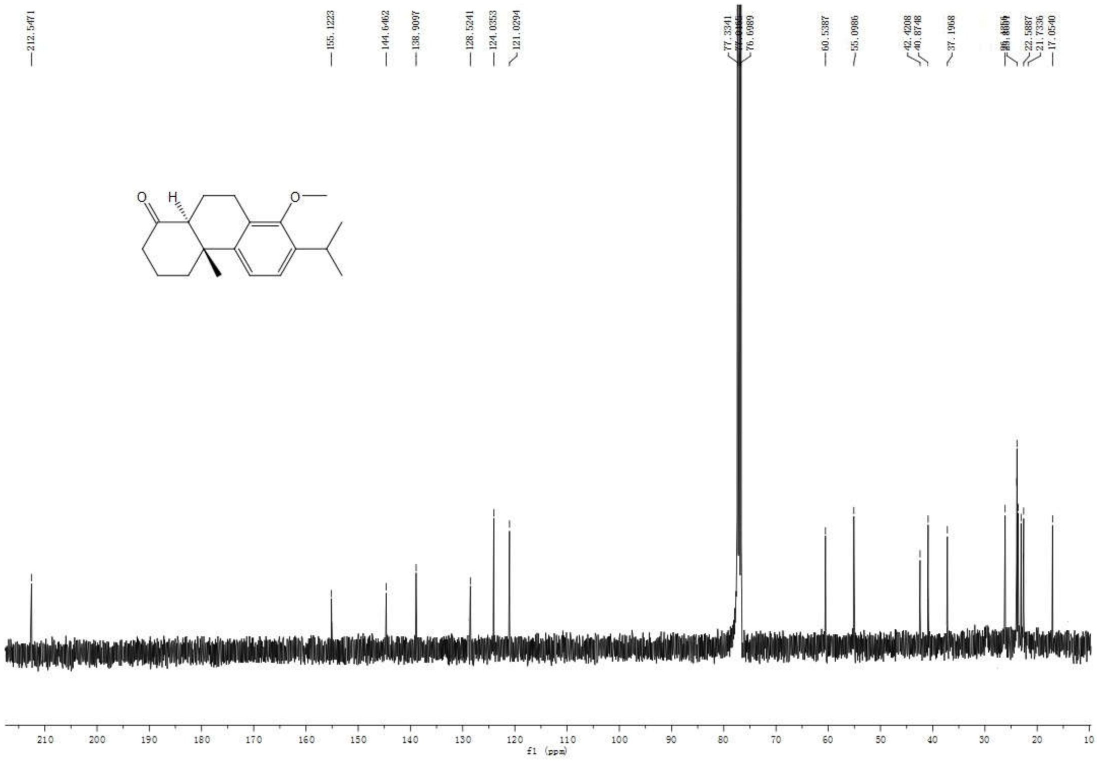

212.5471
155.1223
144.6462
138.9097
128.5241
124.0353
121.0294
77.3341
76.6989
60.5387
55.0986
42.4208
40.8748
37.1968
22.5887
21.7336
17.0540
O
H
O
f1 (ppm)

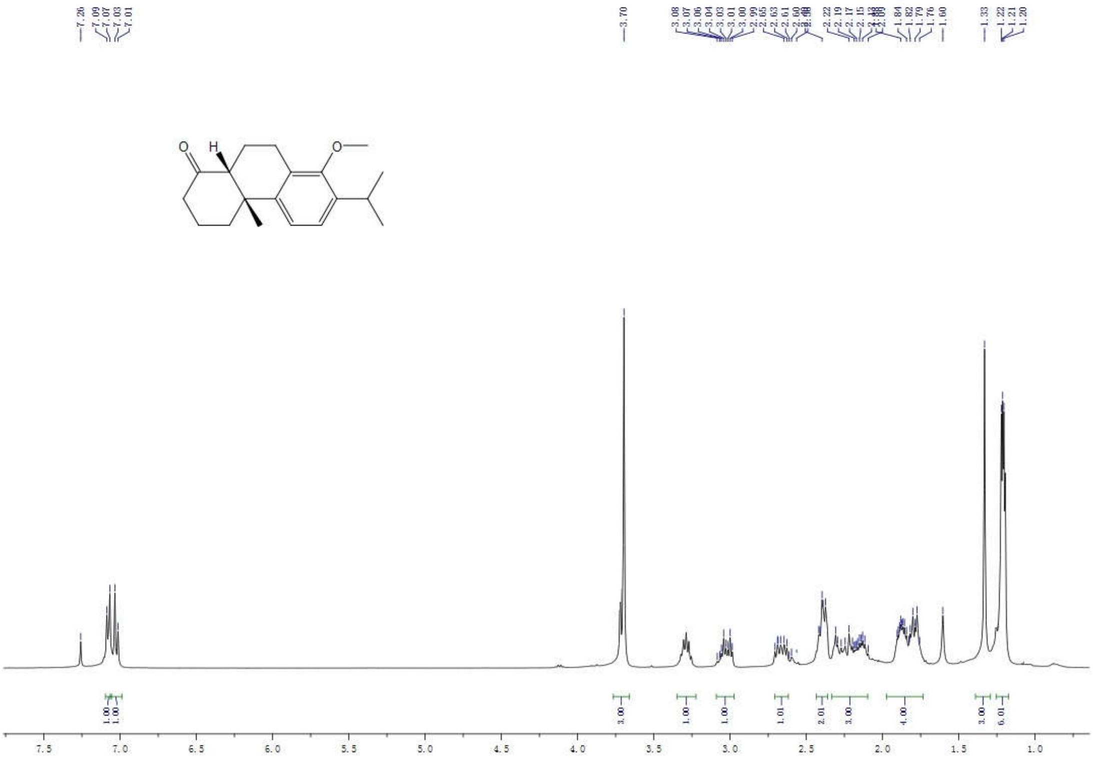
7.26
7.09
7.07
7.03
7.01
3.70
1.84
1.82
1.79
1.76
1.60
1.33
1.22
1.21
1.20
1.00
1.00
3.00
1.00
1.00
1.01
2.01
3.00
4.00
3.00
6.01
7.5
7.0
6.5
6.0
5.5
5.0
4.5
4.0
3.5
3.0
2.5
2.0
1.5
1.0

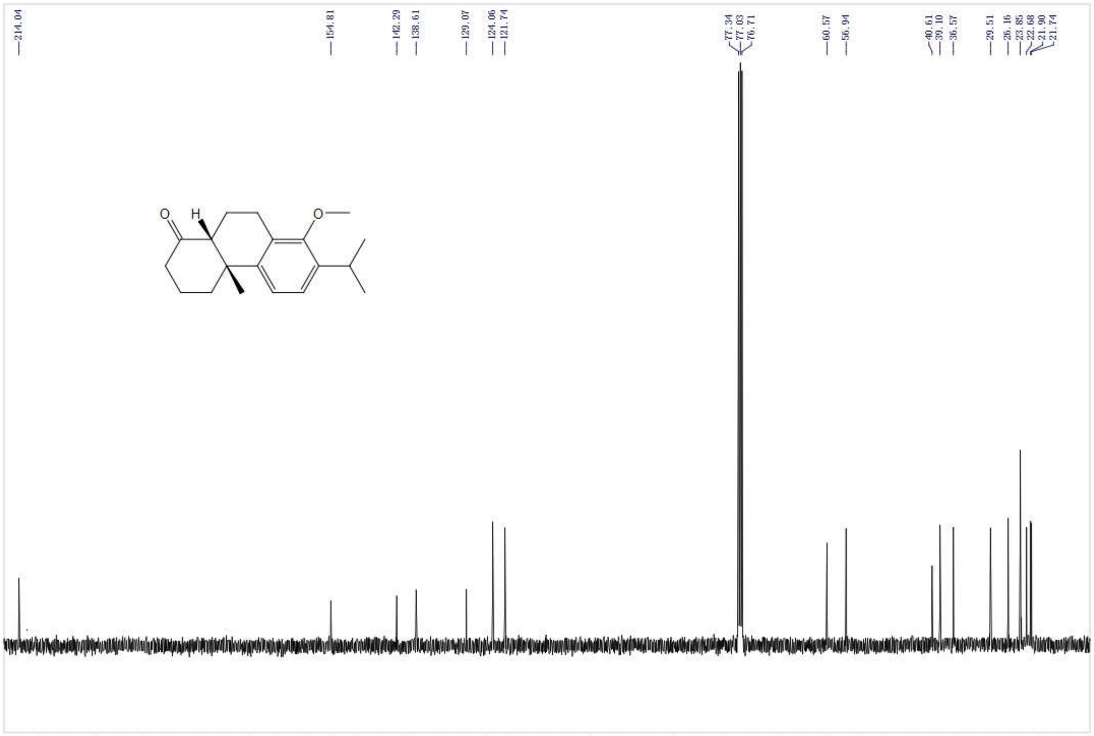
214.04
154.81
142.29
138.61
129.07
124.06
121.74
77.34
77.03
76.71
60.57
56.94
40.61
39.10
36.57
29.51
26.16
23.85
22.68
21.90
21.74
210
200
190
180
170
160
150
140
130
120
110
100
90
80
70
60
50
40
30
20

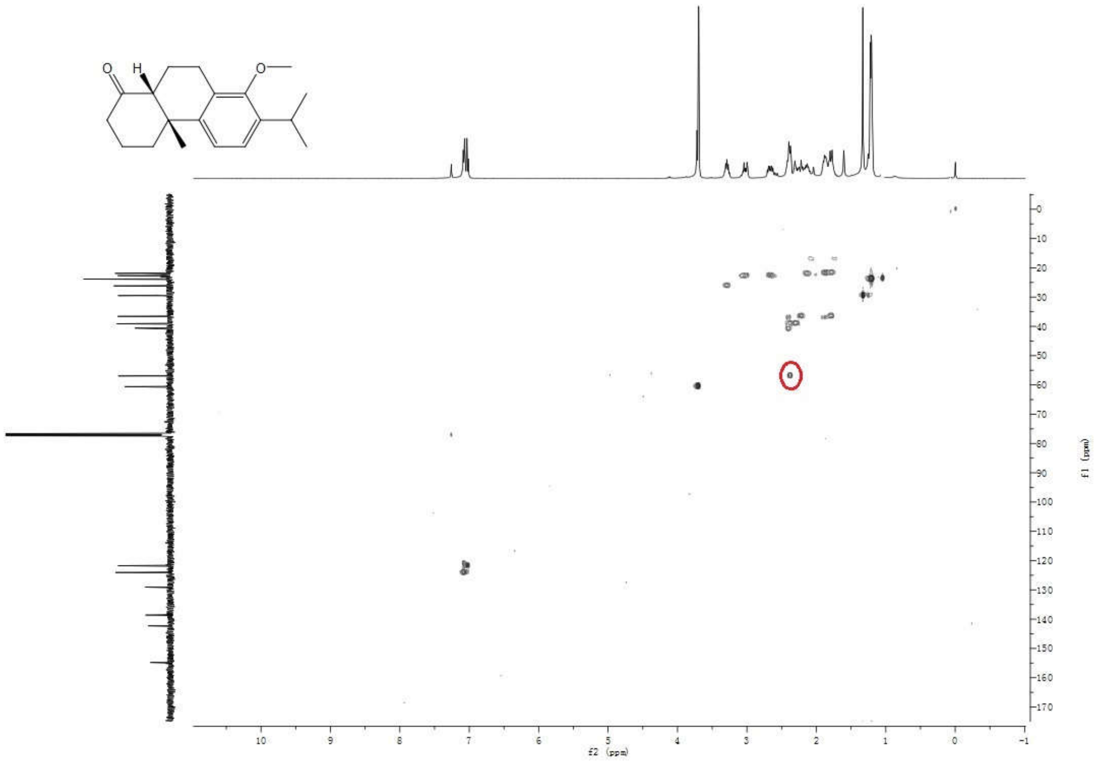

compound noesy

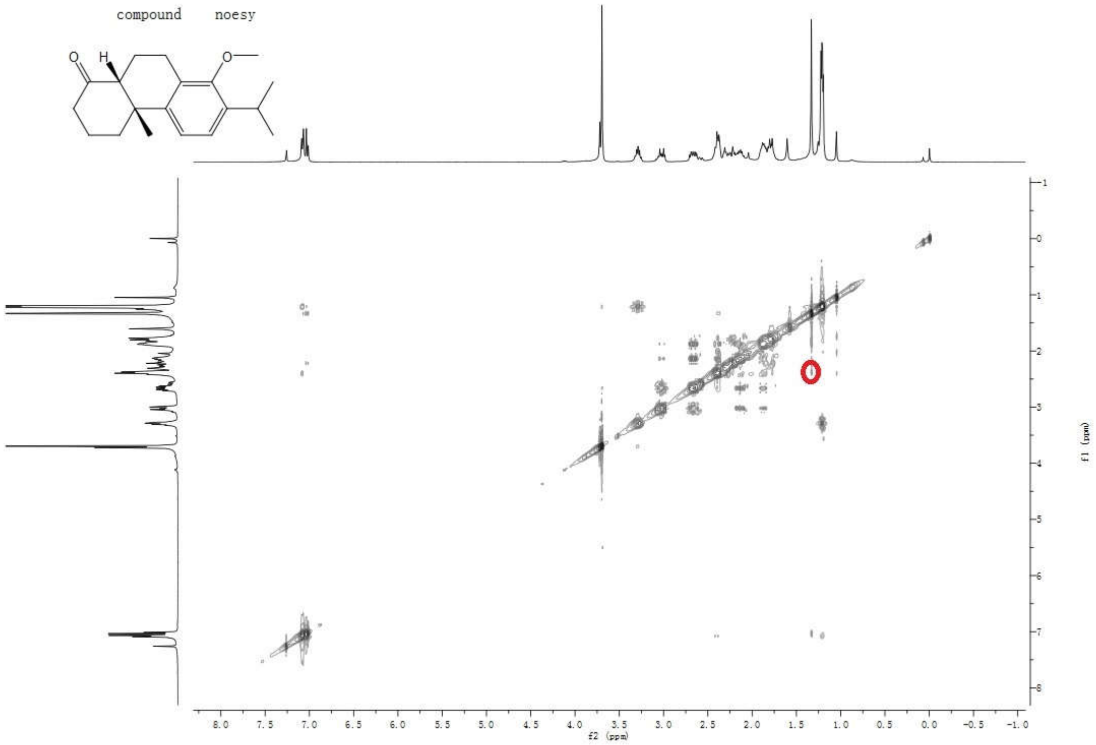

Acquired by : Admin
Sample Name : 131207
Sample ID :
Data File Name : 1207-6BnOIPr-rac-ash-1.lcd
Method File Name : Et-hex(2-98)-1.lcm
Batch File Name :
Report File Name : Eng-sample info and peak table.lcr
Data Acquired : 2013-12-7 14:37:47
Data Processed : 2013-12-7 14:58:29

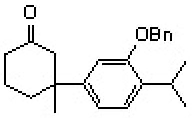

<Chromatogram>

Chromatogram
131207 EALC-20AB\756\1207-6BnOIPr-rac-ash-1.lcd

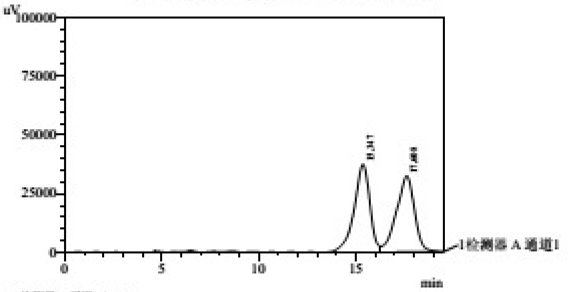

1 检测器 A 通道1 / 254nm

PeakTable

Detector A Ch1 254nm

Peak#	Ret. Time	Area	Height	Area %	Height %
1	15.347	2138813	37532	50.288	53.605
2	17.608	2114291	32484	49.712	46.395
总计		4253105	70016	100.000	100.000

Acquired by : Admin
Sample Name : 131207
Sample ID :
Data File Name : 1207-6BnOIPr-ee-ash-1.lcd
Method File Name : Et-hex(2-98)-1.lcm
Batch File Name :
Report File Name : Eng-sample info and peak table.lcr
Data Acquired : 2013-12-7 15:07:40
Data Processed : 2013-12-7 15:28:11

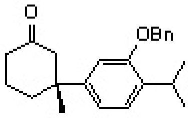

<Chromatogram>

Chromatogram
131207 EALC-20AB\756\1207-6BnOIPr-ee-ash-1.lcd

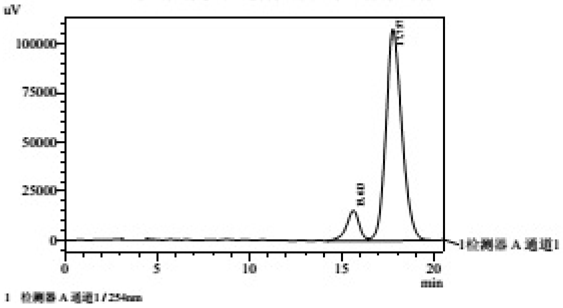

1 检测器 A 通道1 / 254nm

PeakTable

Detector A Ch1 254nm

Peak#	Ret. Time	Area	Height	Area %	Height %
1	15.613	744651	15342	10.249	12.471
2	17.757	6521235	107674	89.751	87.529
总计		7265885	123015	100.000	100.000

Acquired by : Admin
Sample Name : 140118
Sample ID :
Data File Name : 118-666-TM-rac-ash-et.lcd
Method File Name : Et-hex(2-98)-1.lcm
Batch File Name :
Report File Name : Eng-sample info and peak table.lcr
Data Acquired : 2014-1-18 11:11:57
Data Processed : 2014-1-18 15:00:05

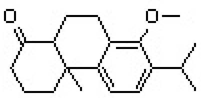

<Chromatogram>

Chromatogram
140118 E:\LC-20AB\756\118-666-TM-rac-ash-et.lcd

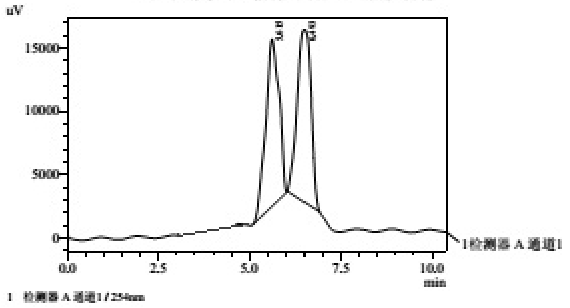

1 检测器 A 通道1 / 254nm

PeakTable

Detector A Ch1 254nm

Peak#	Ret. Time	Area	Height	Area %	Height %
1	5.615	338083	13201	49.689	49.239
2	6.493	342314	13609	50.311	50.761
总计		680397	26811	100.000	100.000

Acquired by : Admin
Sample Name : 140118
Sample ID :
Data File Name : 118-666-TM-ee-ash-et.lcd
Method File Name : Et-hex(2-98)-1.lcm
Batch File Name :
Report File Name : Eng-sample info and peak table.lcr
Data Acquired : 2014-1-18 11:27:38
Data Processed : 2014-1-18 11:46:37

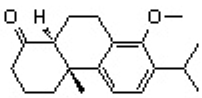

<Chromatogram>

Chromatogram
140118 E:\LC-20AB\756\118-666-TM-ee-ash-et.lcd

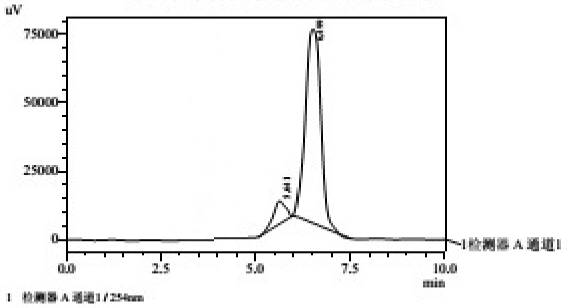

1 检测器 A 通道1 / 254nm

PeakTable

Detector A Ch1 254nm

Peak#	Ret. Time	Area	Height	Area %	Height %
1	5.641	189380	8322	8.646	10.528
2	6.499	2000930	70732	91.354	89.472
总计		2190310	79054	100.000	100.000
